# Systematically Engineering for Efficient Production of 3‐Methyl‐1‐Butanol in *Escherichia coli*

**DOI:** 10.1002/advs.202520521

**Published:** 2026-02-12

**Authors:** Nanfei Geng, Hao Liu, Haolin Han, Meng Meng, Shaojie Wang, Tianwei Tan, Haijia Su

**Affiliations:** ^1^ State Key Laboratory of Green Biomanufacturing National Energy R&D Center for Biorefinery Beijing Key Laboratory of Green Chemicals Biomanufacturing Beijing Synthetic Bio‐manufacturing Technology Innovation Center Beijing University of Chemical Technology Beijing P. R. China

**Keywords:** 3‐methyl‐1‐butanol, *Escherichia coli*, metabolic engineering, semi‐rational design, system optimization

## Abstract

3‐Methyl‐1‐butanol (3‐MB), a promising next‐generation biofuel, has garnered significant interest owing to its superior combustion characteristics and fuel compatibility. However, current 3‐MB biosynthesis faces major challenges, including low production efficiency and severe toxicity‐induced growth inhibition, which significantly limit its industrial feasibility. In this study, we systematically developed an integrated metabolic engineering approach for high‐level 3‐MB production in *Escherichia coli*. Through semi‐rational engineering of the rate‐limiting enzyme dihydroxyacid dehydratase (DHAD), combined with molecular dynamics simulations, we identified and addressed previously unrecognized catalytic bottlenecks. The engineered strain exhibited a 32.3‐fold increase in 3‐MB production, reaching 2.20 g/L in shake‐flask cultures. Subsequent adaptive laboratory evolution further improved strain robustness, while genomic analysis revealed novel regulatory targets for metabolic optimization. In a scaled‐up bioreactor fermentation system, the final strain achieved a record titer of 6.24 g/L, representing the highest reported titer for engineered microbial systems. This work not only establishes a scalable platform for 3‐MB biosynthesis but also provides a modular engineering framework applicable to other advanced biofuels.

## Graphical abstract

An integrated metabolic engineering strategy was established for high‐level 3‐methyl‐1‐butanol biosynthesis in Escherichia coli. Molecular dynamics‐guided semi‐rational engineering of dihydroxyacid dehydratase uncovered and relieved key catalytic bottlenecks, while adaptive laboratory evolution enhanced strain robustness. The optimized strain achieved a record titer of 6.24 g/L in bioreactor fermentation, providing a scalable framework for advanced biofuel production.

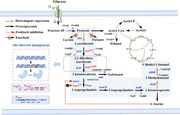

## 1 Introduction

The depletion of non‐renewable energy sources, such as oil and natural gas, coupled with the growing challenges of climate change, has accelerated the search for sustainable biofuels [1, 2]. Among biofuels, higher alcohols have drawn significant interest due to their superior fuel properties and compatibility with existing infrastructure [3]. 3‐Methyl‐1‐butanol (3‐MB) is particularly promising as it offers a high energy density and can be blended with gasoline in any ratio, making it suitable for use in spark‐ignition engines [4, 5]. It also serves as a valuable anti‐knock agent and can be converted into aviation fuels, broadening its potential applications [6]. Beyond fuel applications, 3‐MB is a versatile compound used in the production of resins, coatings, pharmaceuticals, and other industrial products [7].

Microbial production of 3‐MB presents an attractive alternative to conventional chemical synthesis, as it offers several advantages, including mild reaction conditions, reduced environmental impact, and the use of renewable biomass as feedstock [8]. Microbial production of 3‐MB involved four major pathways: (i) the non‐fermentative pathway [9], (ii) the isopentenyl pyrophosphate (IPP)/dimethylallyl pyrophosphate (DMAPP) pathway [10], (iii) the isovaleryl‐CoA pathway [11], and (iv) the photosynthetic pathway [12]. Among these, the non‐fermentative pathway exhibits distinct advantages due to its compatibility with established metabolic processes and its potential for high‐throughput production. This pathway offers promising opportunities for the biosynthesis of 3‐MB by capitalizing on advancements in amino acid synthesis technologies and optimizing anabolic pathways within host cells [13, 14].

Current research has primarily focused on host selection and pathway construction, while systematic breakthroughs in enzyme optimization, metabolic flux remodeling, and tolerance engineering remain lacking, leaving the core metabolic bottlenecks unresolved. Atsumi [9] first constructed a heterologous Ehrlich pathway in *E. coli* by overexpressing the relevant genes from pyruvate to 3‐MB, relieving feedback inhibition, and deleting competing endogenous pathways, achieving a titer of 1.28  g/L. Hammer [15] engineered *Saccharomyces cerevisiae* by deleting *BAT1*, *LEU4*, *LEU9*, and *OAC1*, combined with mitochondrial targeting of a feedback‐insensitive Leu4 alongside Leu1 and Leu2, and additional cytosolic expression of the same enzymes from a single‐copy plasmid, achieving a 3‐MB titer of 1.24 g/L. Vogt [16] employed a combinatorial strategy in *Corynebacterium glutamicum* by overexpressing the keto‐acid degradation pathway while downregulating endogenous branched‐chain transaminase activity, achieving a 3‐MB titer of 2.76 g/L. Su [17] obtained high‐producing BCHAs mutants in *Brevibacterium flavum* via whole‐cell mutagenesis and combinatorial overexpression of *LEU1*, *LEU4*, *kivD*, and *ADH2*, achieving a 3‐MB titer of 0.79 g/L. Despite these important advances, the 3‐MB biosynthetic pathway has yet to be systematically integrated or comprehensively optimized. The key rate‐limiting steps remain insufficiently characterized, production titers are still relatively low, and in‐depth investigations into product toxicity tolerance and host metabolic compatibility remain scarce.

**FIGURE 1:**
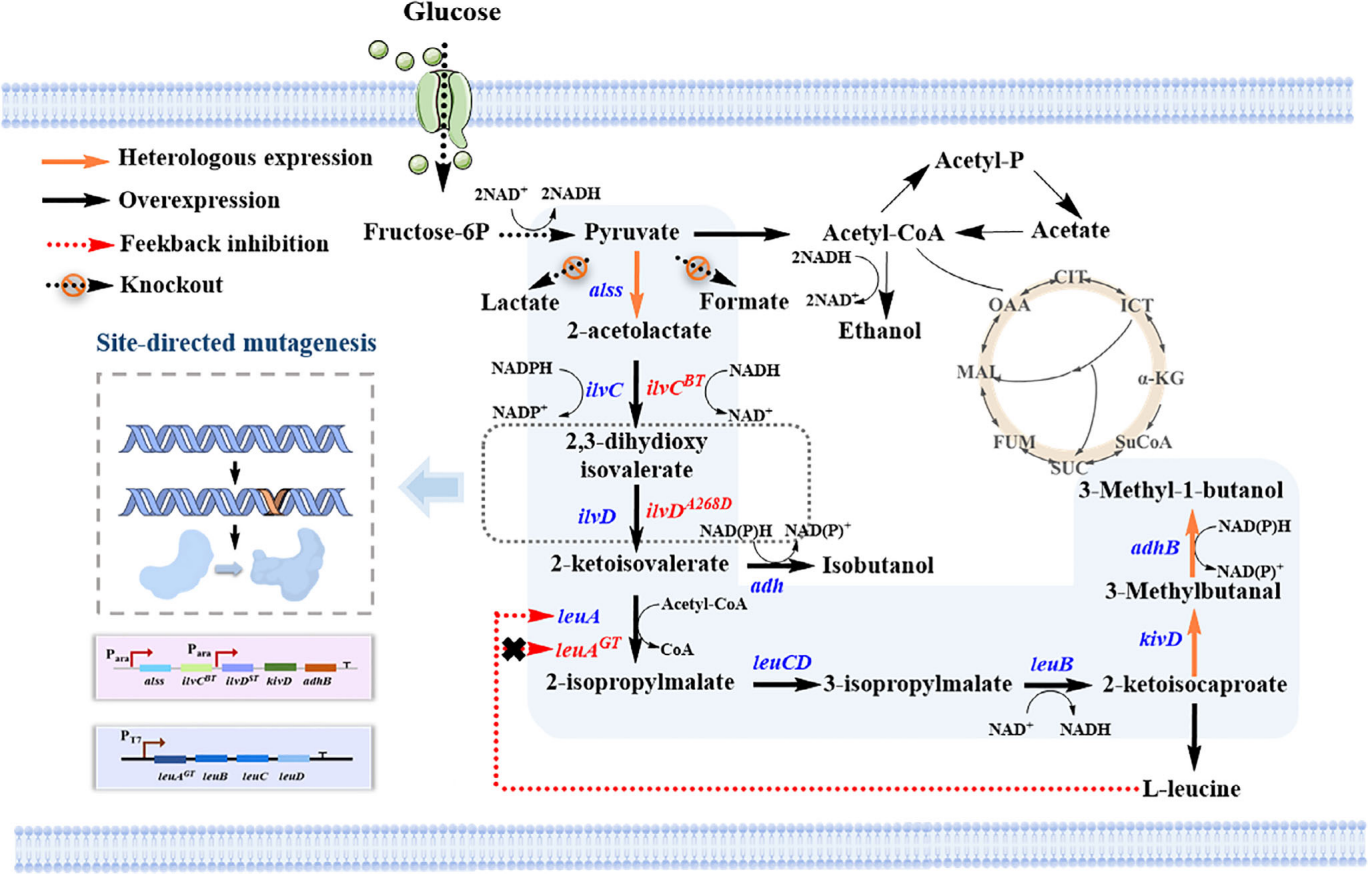
Schematic diagram of the optimized pathway of engineered *E. coli* for high production of 3‐MB. The gene *alss* encodes acetolactate synthase, *ilvC* (*ilvC^BT^*) encodes ketoacid reductoisomerase (mutant), *ilvD* (*ilvD^A268D^*) encodes dihydroxyacid dehydrogenase (mutant), *kivD* encodes ketoacid decarboxylase, *adhB* encodes alcohol dehydrogenase, *leuA* (*leuA^GT^*) encodes 2‐isopropyl malate synthase (mutant), *leuB* encodes 3(β)‐isopropyl malate dehydrogenase, *leuC* encodes isopropyl malate isomerase, and *leuD* encodes 2‐isopropyl malate synthase.

In this study, we developed an integrated and scalable metabolic engineering strategy for high‐level production of 3‐MB in *E. coli* (Figure 1). Systematic pathway rewiring and global flux optimization enabled the identification of previously underappreciated rate‐limiting steps, followed by semi‐rational engineering of the key enzyme dihydroxyacid dehydratase (DHAD). Molecular dynamics simulations were further employed to elucidate key regulatory mechanisms associated with substrate binding and enzyme structural flexibility. The engineered strain produced 2.20 g/L 3‐MB under optimized shake‐flask conditions. To further enhance cellular robustness, adaptive laboratory evolution (ALE) was applied, and whole‐genome sequencing revealed potential mutations associated with product tolerance. As a result, the optimized strain sustained efficient 3‐MB biosynthesis under bioreactor conditions, achieving a titer of 6.24 g/L, which markedly exceeds previously reported levels. Overall, this work establishes a coordinated and industrially relevant platform for microbial 3‐MB production.

## 2 Results

### 2.1 Construction and Optimization of the 3‐MB Biosynthesis Pathway

**FIGURE 2:**
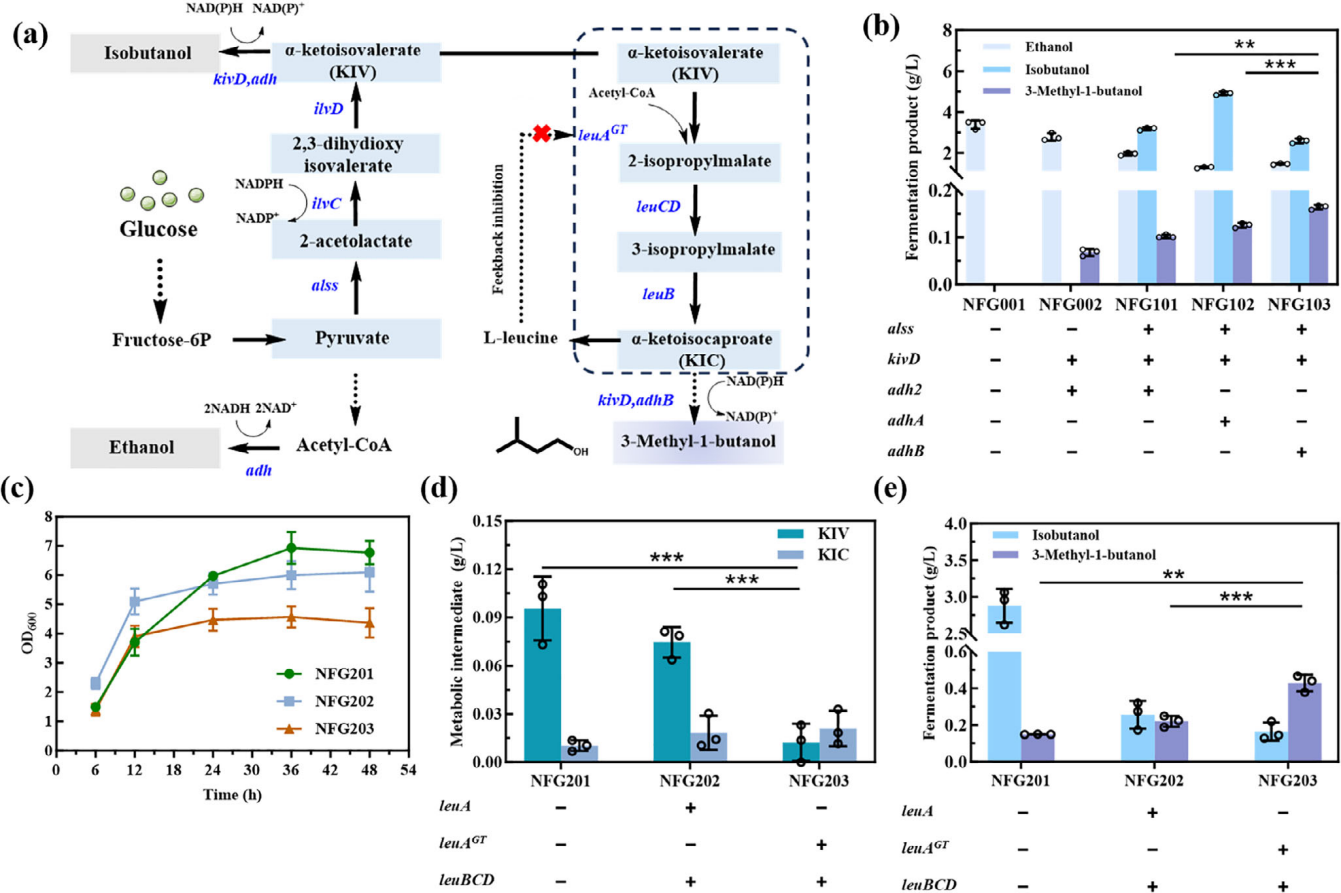
Construction and optimization of the 3‐MB biosynthesis pathway. (a) Schematic illustration of the engineering strategy for *E. coli* 3‐MB biosynthesis through pathway modulation. Dotted lines represent multi‐step reactions, whereas solid lines indicate single‐step conversions. (b) Fermentation products of engineered strains NFG001, NFG002, NFG101, NFG102, and NFG103. (c) Growth curve of engineered strains NFG201, NFG202, and NFG203. (d) Intracellular accumulation of intermediate metabolites in engineered strains NFG201, NFG202, and NFG203. (e) Fermentation products of engineered strains NFG201, NFG202, and NFG203. All experiments were conducted in at least two independent biological replicates. Data are presented as the mean ± s.d. of three biological replicates from a representative experiment. Statistical analysis was performed using a t‐test (two‐tailed, **p<0.01, ***p<0.001). Each experiment was independently performed at least twice, and representative measurements from three biological replicates are presented as mean ± s.d.

To construct a non‐fermentative pathway for 3‐methyl‐1‐butanol (3‐MB) synthesis, we initially introduced a promiscuous α‐keto acid decarboxylase gene (*kivD*) from *Lactococcus lactis* (*L. lactis*), along with an alcohol dehydrogenase gene *adh2* from *Saccharomyces cerevisiae* (*S. cerevisiae*) (Figure 2a). This strain NFG002 successfully produced 68.11 mg/L 3‐MB (Figure 2b). Subsequently, we introduced a heterologous acetolactate synthase gene (*alss*) from *Bacillus subtilis* (*B. subtilis*). This enzyme catalyzes the condensation of two pyruvate molecules to form 2‐acetolactate, and was reported to have a higher affinity for pyruvate than the three native acetohydroxy acid synthase (AHAS) isozymes in *E. coli* BL21, encoded by the *ilvBN*, *ilvGM*, and *ilvIH* genes [9]. Finally, the engineered strain NFG101 achieved a 50.0% increase in 3‐MB production and 3.15 g/L of isobutanol without causing any significant impact on cell growth (Figure 2b; Figures S1–S3).

Alcohol dehydrogenase (ADH) plays a pivotal role in the final catalytic step of 3‐MB biosynthesis, catalyzing the reduction of aldehydes to alcohols. However, it was reported that heterologous expression of *adh2* in *E. coli* can lead to protein misfolding, aggregation, and reduced enzymatic activity, ultimately limiting alcohol production efficiency [14]. Therefore, we evaluated two additional ADHs: *adhA* from *L. lactis* and *adhB* from *Zymomonas mobilis* (*Z. mobilis*), resulting in the construction of strains NFG102 and NFG103, respectively. Among the tested variants, NFG103 demonstrated the highest catalytic preference for 3‐MB, resulting in a 3‐MB titer of 0.16 g/L. In contrast, strain NFG102, expressing *adhA* from *L. lactis*, exhibited a high preference for isobutyraldehyde over 3‐MB, resulting in 0.13 g/L 3‐MB but a significantly higher isobutanol titer of 4.91 g/L (Figure 2b).

While the introduction of non‐fermentative pathways in *E. coli* enabled the biosynthesis of 3‐MB, the titer remained relatively low. During fermentation, a significant accumulation of isobutanol was observed, suggesting that carbon flux was being diverted toward undesired by‐products. Therefore, we further overexpressed the *leuABCD* operon, which is responsible for converting α‐ketoisovalerate (KIV) to α‐ketoisocaproate (KIC), a crucial intermediate in the production of 3‐MB. The resulting strain NFG202 exhibited a 47.3% higher 3‐MB production titer, while significantly reducing isobutanol accumulation from 2.88 g/L to 0.26 g/L.

Further investigation revealed that 2‐isopropylmalate synthase (IPMS), encoded by *leuA*, is subject to feedback inhibition by leucine, which limits the efficient conversion of α‐ketoisovalerate (KIV) to α‐ketoisocaproate (KIC) and consequently constrains the metabolic flux toward 3‐MB biosynthesis. Quantitative analysis of leucine accumulation in the engineered strain NFG202 showed that its concentration reached approximately 0.24 g/L (1.83 mM), which greatly exceeds the reported threshold for significant IPMS feedback inhibition. This result indicates that leucine exerts substantial inhibitory pressure on IPMS during fermentation (Figure S4). Relieving IPMS feedback inhibition effectively sustains carbon flux toward KIC, thereby significantly enhancing 3‐MB production. To overcome this regulatory bottleneck, we introduced a feedback‐insensitive variant of the *leuA* gene (*leuA^GT^*, G462D), which bypassed leucine‐mediated inhibition [18]. This mutation enhanced IPMS activity, improving KIV conversion efficiency (Figure 2d). Although there was some impact on cell growth (Figure 2c; Figure S5), the strain NFG203 achieved a remarkable 67.6% increase in 3‐MB production, reaching a final titer of 0.43 g/L. (Figure 2e).

### 2.2 Cofactor Regulation and Fine‐Tuned Expression of Key Genes

**FIGURE 3:**
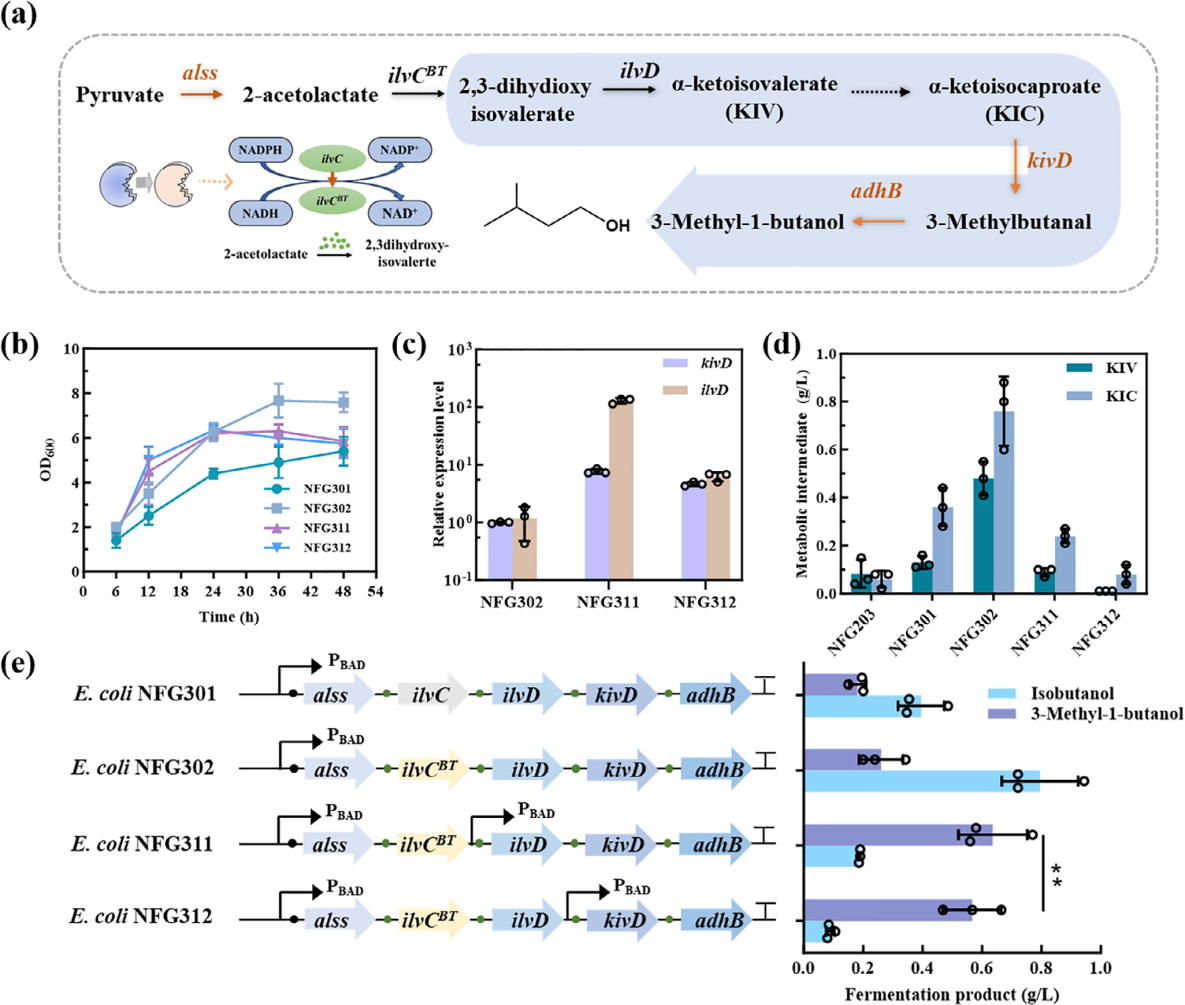
Cofactor regulation and fine‐tuned expression increase the production of 3‐MB in *E. coli*. (a) Schematic diagram of cofactor regulation and gene‐enhanced expression. The dotted lines indicate multiple steps and the solid lines a single step. The orange line indicates the heterologous pathway, and the black line indicates the endogenous pathway. (b) Growth of engineered *E. coli* strains NFG301, NFG302, NFG311 and NFG312. (c) Relative expression levels of key genes in engineered *E. coli* strains. (d) Intracellular accumulation of intermediate metabolites in engineered strains NFG203, NFG301, NFG302, NFG311 and NFG312. (e) Fermentation products of engineered *E. coli* strains NFG301, NFG302, NFG311 and NFG312. Statistical analysis was performed using a t‐test (two‐tailed, **p<0.01). Each experiment was independently performed at least twice, and representative measurements from three biological replicates are presented as mean ± s.d.

To push more carbon flux toward 3‐MB synthesis, we overexpressed the endogenous NADPH‐dependent acetolactate isomeroreductase gene (*ilvC*) and the dihydroxy acid dehydratase gene (*ilvD*), which catalyze the conversion of 2‐acetolactate to KIV (Figure 3a). However, the engineered strain NFG301 showed a significant decrease in 3‐MB titer, reducing to 0.18 g/L, likely due to a corresponding reduction in cell growth (Figure 3b; Figure S6). This decline in biomass may be attributed to imbalances in intracellular redox homeostasis, which is essential for maintaining both metabolic activity and cell viability (Table S4).

Recognizing the importance of redox balance, we overexpressed a modified NADH‐dependent *ilvC^BT^* gene (A71S:R76D:S78D:Q110V) to alleviate the NADPH imbalance caused by the endogenous *ilvC* [19]. Consequently, the biomass of the engineered strain NFG302 increased by 62% compared to strain NFG301 (Figure 3b), but this improvement in cell growth did not lead to a significant increase in 3‐MB production. Further analysis revealed that KIC and KIV accumulated at 0.48 g/L and 0.76 g/L, respectively (Figure 3d), indicating a bottleneck in the downstream conversion process.

To further optimize 3‐MB production, we constructed dual‐promoter expression cassettes and fine‐tuned the expression levels of key genes involved in the 3‐MB synthesis. Among these engineered strains, strain NFG311 showed the highest titer of 0.64 g/L 3‐MB, representing an 8.4‐fold increase compared to the initial strain (Figure 3e). It is noteworthy that upregulation of the *ilvD* gene significantly enhanced 3‐MB production in strain NFG311, highlighting its key role in driving the biosynthetic flux. Although both *kivD* and *adhB* were highly expressed in NFG311 and NFG312 (Figure 3c), only NFG311 exhibited markedly improved 3‐MB titers. These results indicate that the reaction catalyzed by DHAD may represent a critical rate‐limiting step in the overall 3‐MB biosynthetic pathway.

### 2.3 Rational DHAD Design Unlocks High‐Efficiency 3‐MB Biosynthesis

Although we found that the *ilvD* gene might play a pivotal role in the synthesis of 3‐MB, this gene had not received significant attention in prior research. To address this gap, we employed site‐directed mutagenesis methods to rationally design the dihydroxyacid dehydratase (DHAD) encoded by the *ilvD* gene [20].

By examining the catalytic mechanism and the three‐dimensional structure of the enzyme predicted by AlphaFold2, the substrate 2,3‐dihydroxy isovalerate was docked into DHAD [21]. Based on the receptor‐ligand structure of DHAD and its substrate, all the amino acid residues within 3 Å of the substrate 2,3‐dihydroxy isovalerate were regarded as potential mutation sites, and these residues were respectively mutated to ALA. The top 5 residues with the highest mutation energy were considered to be key residues, which were CYS195, MET85, LEU164, PHE199, and ALA268. These 5 key residues were further performed in single‐site virtual saturation mutagenesis calculations. The mutation residues with the lowest mutation energy were considered as those that might enhance the affinity between DHAD and its substrate. The effects of the designed mutations were subsequently verified through experimental testing, with the specific mutation sites detailed in Table S5.

**FIGURE 4:**
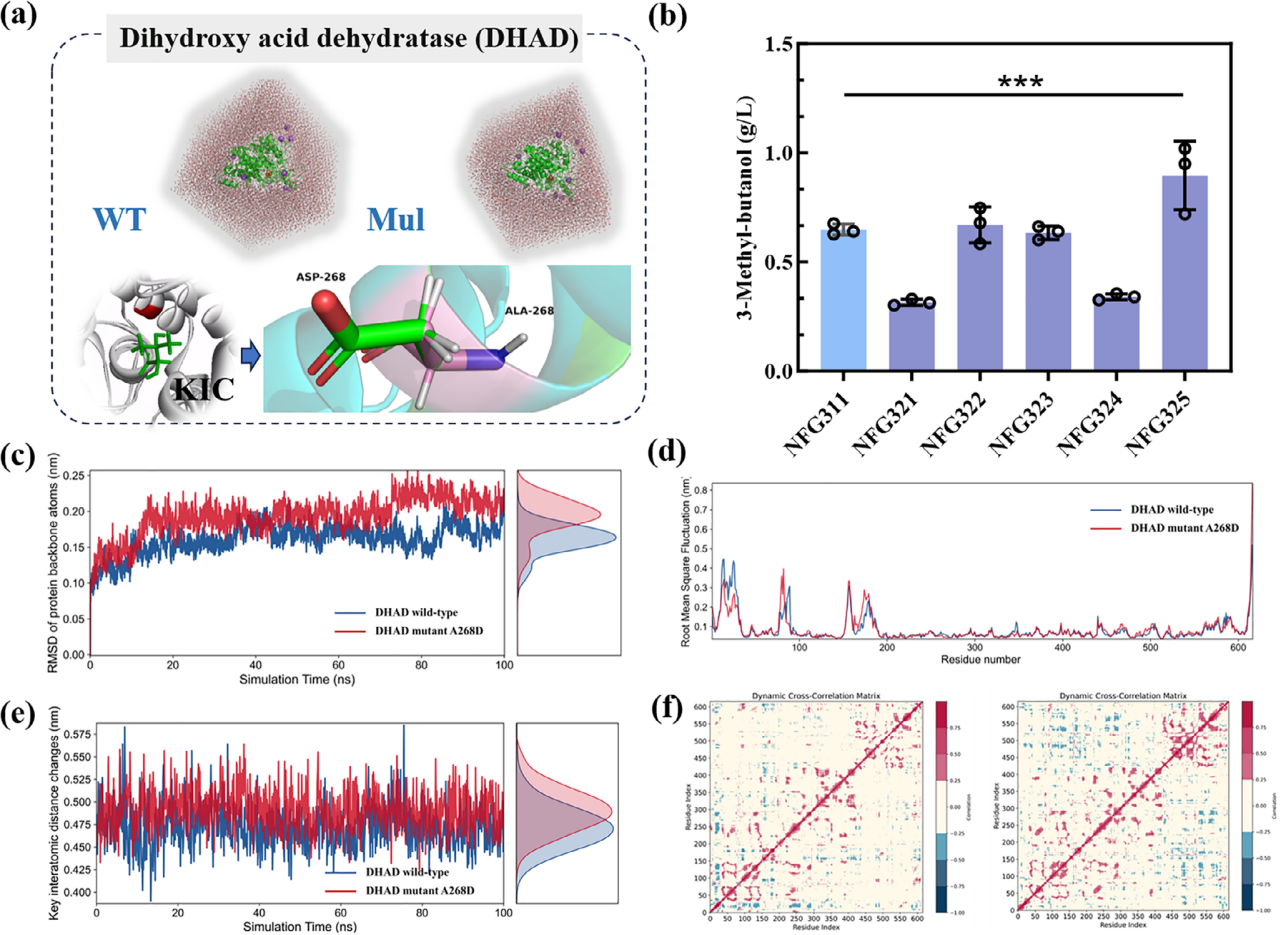
Effect of rational modification of dihydroxyacid dehydratase on the synthesis of 3‐MB. (a) Dihydroxyacid dehydratase (DHAD) and A268D mutant. (b) 3‐MB titers of engineered *E. coli* strains NFG311, NFG321, NFG322, NFG323, NFG324 and NFG325. Statistical analysis was performed using a t‐test (two‐tailed, ***p<0.001). (c) The RMSD values during a 100 ns molecular dynamics simulation. (d) The RMSF values calculated during 100 ns. (e) Comparison of key atomic distance variations. (f) Dynamic cross‐correlation matrix comparison between the wild‐type protein (left) and the DHAD mutant (right). Statistical analysis was performed using a t‐test (two‐tailed, ***p<0.001). Each experiment was independently performed at least twice, and representative measurements from three biological replicates are presented as mean ± s.d.

We overexpressed these DHAD mutants in strain NFG311, creating a series of strains NFG321‐NFG325 to evaluate their impact on 3‐MB synthesis. Among all the engineered strains, strain NFG325, which carried the A268D mutation (Figure 4a), demonstrated the highest titer of 3‐MB, achieving 0.90 g/L. This represents a significant 40.1% increase compared to the control strain NFG311, which overexpressed the wild‐type *ilvD* gene, while maintaining comparable biomass. (Figure 4b; Figure S7). The kinetic parameters of the enzyme showed that the specific activity of the DHAD mutant (A268D) was significantly increased by 42.9%, and the difference in *k*_cat_/*K*_m_ further demonstrated the enhanced catalytic efficiency (Table 1).

**TABLE 1:** Kinetic parameters of DHAD mutant.

	Specific Activity (U/mg)	*K*_m_ (µM)	*k*_cat_ (min^−1^)	*k*_cat_/*K*_m_ (min^−1^ µM^−1^)
DHAD (*ilvD*)	8.08 ± 0.32	62.8 ± 0.6	0.702 ± 0.013	0.011
DHAD mutant (*ilvD^A268D^*)	11.55 ± 0.43	63.3 ± 0.4	0.936 ± 0.011	0.015

Molecular dynamics (MD) simulation was utilized to elucidate the molecular mechanism underlying the enhanced catalytic performance of the A268D mutant. To quantitatively assess the binding affinity differences, binding free energy calculations were performed using gmx_MMPBSA based on the last 25 ns of the MD trajectories (Table S6). The results revealed that the A268D mutant exhibited significantly stronger binding affinity (ΔG = −14.30 ± 3.16 kcal/mol) compared to the wild‐type DHAD (ΔG = −8.01 ± 2.72 kcal/mol). Energy decomposition analysis showed that this enhancement primarily stems from dramatically improved electrostatic interactions (ΔEEL changed from +25.76 to −10.86 kcal/mol), indicating that the negatively charged aspartate residue at position 268 creates a favorable electrostatic environment within the active site.

Root mean square deviation (RMSD) analysis revealed that the A268D mutant exhibited more constrained conformational fluctuations, with backbone atom RMSD values consistently lower than those of the wild‐type DHAD throughout the simulation (Figure 4c). This enhanced structural stability directly correlates with the 33% increase in catalytic turnover rate (*k*_cat_: 0.702 → 0.936 min^−^^1^) observed experimentally, suggesting that reduced conformational flexibility maintains the optimal geometry of the active site during catalysis. Root mean square fluctuation (RMSF) analysis showed distinct dynamic features in the N‐terminal region and around residues 100 and 170 in the mutant DHAD, while the C‐terminal region maintained high flexibility, highlighting local conformational differences between the two enzymes (Figure 4d).

The enhanced binding affinity observed computationally (ΔG = −14.30 vs −8.01 kcal/mol) provides valuable insights into the thermodynamic stability of the enzyme‐substrate complex. It should be noted that enzyme‐substrate interactions involve complex regulatory mechanisms at multiple levels, and the relationship between computational binding energies and experimental kinetic parameters may reflect different aspects of the catalytic process. The *K*_m_ values, which represent the dynamic equilibrium under steady‐state conditions, showed minimal variation between wild‐type and mutant enzymes (62.8 vs 63.3 µM), suggesting that the mutation primarily influences the catalytic conversion step rather than initial substrate recognition. This is consistent with the substantial improvement in catalytic turnover (*k*_cat_ increased by 33%) and overall catalytic efficiency (*k*_cat_/*K*_m_ improved by 36%), indicating that the A268D mutation optimizes the enzyme's ability to convert bound substrate to product while maintaining effective substrate binding capacity. Furthermore, analysis of the distance between the centroid of Fe2‐S2 cluster and the substrate during MD simulations demonstrated that the mutant DHAD exhibited smaller fluctuations in key atomic distances, indicating a more stable conformational ensemble (Figure 4e). This enhanced stability of the catalytic center is consistent with the improved electron transfer efficiency required for the dehydration reaction, directly supporting the observed increase in *k*_cat_. Dynamic cross‐correlation matrix (DCCM) comparison revealed substantial changes in correlated motions induced by the A268D mutation, with the mutant showing altered communication networks throughout the protein structure (Figure 4f). This global reorganization of the dynamic network suggests that the single‐point mutation modulates protein function through long‐range allosteric effects, optimizing the conformational states required for efficient catalysis.

In summary, the A268D mutation enhances DHAD catalytic performance through a dual mechanism: (1) electrostatic stabilization of the enzyme‐substrate complex, as evidenced by improved binding free energy, and (2) conformational optimization that maintains active site geometry during catalysis, resulting in significantly improved catalytic turnover while preserving substrate binding affinity.

### 2.4 Knockout of Competing Pathways to Maximize Production

**FIGURE 5:**
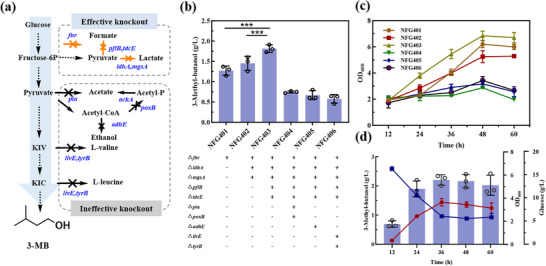
Knockout of competing pathways improves 3‐MB production in engineered *E. coli*. (a) Knockout pathway for byproducts in *E. coli*. KIV, α‐ketoisovalerate; KIC, α‐ketoisocaproate; 3‐MB, 3‐methyl‐1‐butanol. (b) 3‐MB titers of engineered *E. coli* strains NFG401‐NFG406. (c) Cell growth of engineered *E. coli* strains NFG401‐NFG406. (d) Optimization of fermentation conditions to improve 3‐MB production in engineered *E. coli* NFG403. Statistical analysis was performed using a t‐test (two‐tailed, ***p<0.001). Each experiment was independently performed at least twice, and representative measurements from three biological replicates are presented as mean ± s.d.

Despite the remarkable improvement in 3‐MB production in strain NFG325, significant accumulation of byproducts such as lactate, formate, ethanol, and isobutanol was observed (Figure S8). This indicates that further optimization of the metabolic flux is necessary. To address this, we first knocked out the *fnr* gene, a critical regulator of the cellular respiratory system in *E. coli*. We then disrupted the *ldhA*, *mgsA*, *pflB*, *tdcE*, *pta*, *poxB*, *adhE*, *ilvE* and *tyrB* genes (Figure 5a), which are associated with the synthesis of byproducts such as lactate and formate, to create a series of gene knockout strains [22].

Notably, the knockout of the genes *fnr*, *ldhA*, *mgsA*, *pflB*, and *tdcE* resulted in a significant increase in the yield of 3‐MB, accompanied by a sharp decrease in the levels of lactate and formate (Figure S9). The highest titer of 3‐MB of 1.81 g/L was detected in the NFG403 strain, which increased by 25.6‐fold compared with the original strain NFG002 (Figure 5b). To further diminish competitive pathways, we subsequently targeted the knockout of genes involved in the biosynthesis of ethanol, acetate, and amino acids. However, the engineered strains NFG404, NFG405, and NFG406 exhibited significant growth defects (Figure 5c), accompanied by a marked reduction in 3‐MB production, while the titers of isobutanol and ethanol followed a similar trend and remained at very low levels (Figure S10).

The fermentation process of engineered strains is influenced by numerous factors, including fermentation mode, culture medium composition, inducer concentrations, and fermentation conditions, all of which directly impact microbial growth and the yield of metabolic target products [23]. Effective regulation of the microbial fermentation process is crucial for optimizing substrate conversion rates [24]. Through orthogonal experiments (Figure S11), we identified optimal conditions, including a balanced carbon‐to‐nitrogen ratio, suitable inducer levels, and a controlled oxygen supply.

Under the optimized conditions, the maximum 3‐MB titer reached 2.20 g/L at 36 h, representing a 20.5% increase compared with the pre‐optimization level and a 31.4‐fold improvement over the parental strain NFG002(Figure 5d). Meanwhile, isobutanol accumulation, which was pronounced prior to optimization, was markedly reduced to approximately 0.3 g/L, indicating that carbon flux was more effectively redirected toward the 3‐MB biosynthetic pathway rather than competing byproduct routes. Under optimized microaerobic conditions, other byproducts exhibited distinct dynamic profiles. Acetate levels gradually decreased during fermentation, whereas lactate showed a moderate accumulation at later stages, reflecting coordinated regulation of carbon flux distribution and intracellular redox balance under controlled oxygen supply (Figure S12). Overall, condition optimization effectively promoted carbon allocation toward 3‐MB biosynthesis while alleviating excessive accumulation of major competing byproducts. As a result, the final 3‐MB yield reached 0.16 g/g glucose, with a production rate of 0.061 g/L·h. To the best of our knowledge, this represents the highest reported 3‐MB production achieved by engineered *E. coli* systems to date.

### 2.5 Adaptive Laboratory Evolution Enhances Strain Tolerance

In addition to pathway engineering modifications, product toxicity represents a critical limitation in microbial 3‐MB production, as cell growth is already severely inhibited at the concentration of 1.5 g/L 3‐MB (Figure S13). As an organic solvent, 3‐MB tends to partition into biological membranes, increasing their fluidity and altering their structure, which severely impairs membrane function. Due to the increased proton permeability of the cytoplasmic membrane and inhibition of membrane ATPase, cells lose the ability to maintain internal pH, while also suffering losses of intracellular molecules such as proteins, RNA, and ATP, as well as a severe hindrance in glucose uptake [25, 26].

**FIGURE 6:**
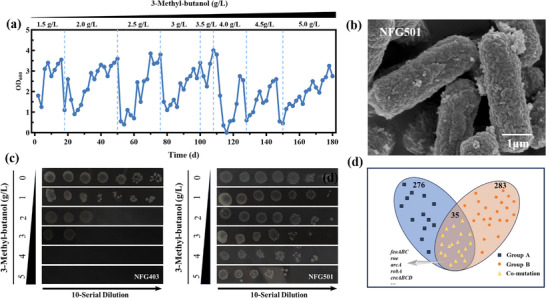
Adaptive laboratory evolution of the strain. (a) Changes in OD values of *E. coli* during 180‐day adaptive evolution with increasing 3‐MB concentrations. (b) Cell morphology of *E. coli* NFG501 under 5 g/L 3‐MB. Images were taken after 6 h of cultivation in the LB medium containing 5 g/L 3‐MB. (c) Strains *E. coli* NFG 403 and NFG501 were spotted on LB plates at different 3‐MB concentrations. (d) Genome re‐sequencing of independently domesticated strains with cross mutations. Group A and Group B are two groups of strains that have undergone ALE independently and all cells were cultured following the experimental procedures.

To this end, we employed an adaptive laboratory evolution (ALE) strategy, passing the high‐producing 3‐MB strain through gradually increasing 3‐MB concentrations over 180 days (Figure 6a). The resulting high‐tolerant strain, NFG501, effectively overcame the inhibitory effect of 3‐MB toxicity on cell growth (Figure 6c), and maintained intact cell morphology and activity even at the lethal concentration of 5 g/L under standard conditions (Figure 6b). Whole‐genome resequencing of NFG501 revealed that the enhancement of 3‐MB tolerance was associated with genes related to membrane function, amino acid transport, sugar transport, and stress response (Table S7).

Therefore, we identified a series of insertion and deletion (InDel) mutations that may contribute to the enhanced 3‐MB tolerance phenotype, suggesting that functional disruption or structural alteration of specific genes could underlie the observed adaptation. Notably, some genes, such as *arcA*, *robA* and *feoA* [27, 28, 29], have been previously validated in the literature to be associated with n‐butanol tolerance, further supporting their potential role in 3‐MB adaptation. Additional affected loci include the *creABCD* operon, regulated by the cAMP receptor protein (CRP) and central to global carbon catabolite repression, as well as *galS* and *mglAB*, which are involved in galactitol uptake and metabolism, indicating a potential rewiring of sugar transport pathways. In addition, the *rne* gene is associated with RNA metabolism and translation, participating in RNA decay, ribosome modification, and translational control. Disruption of this gene may reprogram gene expression under solvent stress, thereby alleviating the translational burden and maintaining protein homeostasis.

The above targets were further validated through reverse metabolic engineering, these experiments demonstrate that the contributions of *robA* and *creABCD* to the tolerant phenotype are particularly prominent, highlighting the central role of global transcriptional regulation and stress response networks in 3‐MB adaptation (Figure S14). Specifically, *robA*, as a global transcriptional regulator, may enhance membrane stability and reduce oxidative stress, while *creABCD*, which governs global carbon metabolism, suggests that carbon flux redistribution is also critical for tolerance. Collectively, these findings not only reveal a previously underappreciated role of post‐transcriptional and global regulatory mechanisms in solvent adaptation, but also establish these loci as strategic targets for engineering robust 3‐MB‐tolerant strains, providing a theoretical basis for future metabolic engineering optimization.

### 2.6 Fed‐Batch Fermentation in a 5 L Bioreactor

**FIGURE 7:**
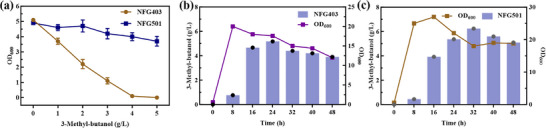
Fed‐batch fermentation in a 5 L bioreactor for 3‐MB production. (a) Effects of different 3‐MB concentration gradients on the growth of engineered *E. coli* strains NFG403 and NFG501. (b) Fermentative production of 3‐MB by engineered *E. coli* strain NFG403 in a bioreactor. (c) Fermentative production of 3‐MB by engineered *E. coli* strain NFG501 in a 5L bioreactor. All cells were cultured according to the experimental procedures.

To evaluate the production capacity of the engineered strains under controlled conditions, fed‐batch fermentation was performed in a 5 L bioreactor. The high‐producing and tolerant strain NFG501 was compared with the conventional strain NFG403 to assess improvements in 3‐MB synthesis and tolerance. The results demonstrated that NFG501 exhibited both higher 3‐MB tolerance and significantly enhanced production, achieving a titer of 6.24 g/L (Figure 7), which corresponds to a 20.5% increase over NFG403 and represents the highest 3‐MB titer reported to date (Table 2). This improvement highlights the effectiveness of the strain engineering strategy and suggests that the modifications successfully optimized the metabolic pathway toward higher 3‐MB yield while maintaining robust cellular performance under inhibitory conditions.

**TABLE 2:** Relevant characteristics of different microbial strains engineered to produce 3‐methyl‐1‐butanol.

Host	Modification methods/ways	Titer (g/L)	Yield (g/g glucose)	Productivity (mg/L/h)	Refs.
*Ralstonia eutropha* LH74D	Overexpression of non‐fermentative pathway genes and disruption of polyhydroxybutyrate biosynthetic genes	0.57	0.11	4.8	[30]
*Brevibacterium flavum* VLICL‐52	Overexpression of the leucine pathway and non‐fermentative pathway genes	0.79	—	10.8	[17]
*C. glutamicum* MV‐KICF1	Overexpression of α‐keto acid decarboxylase and reduction of endogenous branched‐chain amino acid transaminase activity	2.76	0.10	57.6	[16]
*S. cerevisiae*	Deletion of *BAT1* and *ALD6*, overexpression of *ARO10* and *ADH2*, along with the overexpression of valine and leucine pathway genes	0.77	0.0076	7.98	[31]
*S. cerevisiae* MC5b	Overexpression of *ARO10*, *ADH2*, *OAC1*, and valine and leucine pathway genes	0.56	0.014	7.79	[32]
*S. cerevisiae* SHy 252	Deletion of *BAT1*, *LEU4*, *LEU9*, and *OAC1*, and overexpression of the feedback‐insensitive mutants *Leu1* and *Leu2*	1.24	0.012	25.8	[15]
*Pichia pastoris* PP506	Overexpression of the leucine pathway and non‐fermentative pathway genes, and deletion of the gene *PDC1*	0.19	0.002	2.65	[33]
*Ralstonia eutropha*	Disruption of polyhydroxybutyrate biosynthetic genes *phaC1*, *phaA*, *phaB1*, *phaB2*, and *phaC2*; Overexpression of the leucine pathway and non‐fermentative pathway genes	0.57	—	—	[34]
*E. coli* JCL260	Expression of non‐fermentative pathway genes and knockout of the *ilvE* and *tyrB* genes	1.28	0.11	45.7	[18]
*E. coli* KG9	Overexpression of *NudB*, *NemA*, and IPP/DMAPP pathway genes	0.30	0.03	6.25	[35]
*E. coli* XX03	Overexpression of the IPP/DMAPP pathway genes and isovaleryl‐CoA pathway genes, and deletion of the genes *adhE*, *ldhA* and *frdBC*	0.08	0.008	2.24	[11]
*E.coli* NFG403 (shake‐flask)	Overexpression of the leucine pathway and non‐fermentative pathway genes, optimization of pathway genes, and fine‐tuning of pathway expression levels	2.02	0.09	86.7	This study
*E.coli* NFG501 (fed‐batch)	Tolerant chassis via ALE, global pathway optimization, and 5 L fed‐batch fermentation	6.24	0.035	195.0	This study

Interestingly, the production kinetics of the two strains differed notably. NFG501 reached its maximum 3‐MB titer at 36  h, whereas NFG403 peaked much earlier at 24  h (Figure 7b, c). This shift indicates that the prolonged adaptive evolution applied to NFG501 not only enhanced overall production but also extended the duration of active metabolite synthesis, allowing more effective utilization of available carbon sources. Notably, ethanol was produced at low levels but showed no stable accumulation during the fed‐batch fermentation, whereas isobutanol accumulation in NFG501 was substantially reduced compared with NFG403. Furthermore, the overall accumulation of byproducts was markedly reduced, indicating that carbon flux was more effectively channeled toward 3‐MB biosynthesis (Figure S15). The combination of improved tolerance, sustained cellular activity, and efficient carbon utilization may be attributed to more efficient flux through the engineered pathway and enhanced cellular resilience, providing valuable insights for further optimization of industrial‐scale 3‐MB fermentation.

## 3 Discussion

**FIGURE 8:**
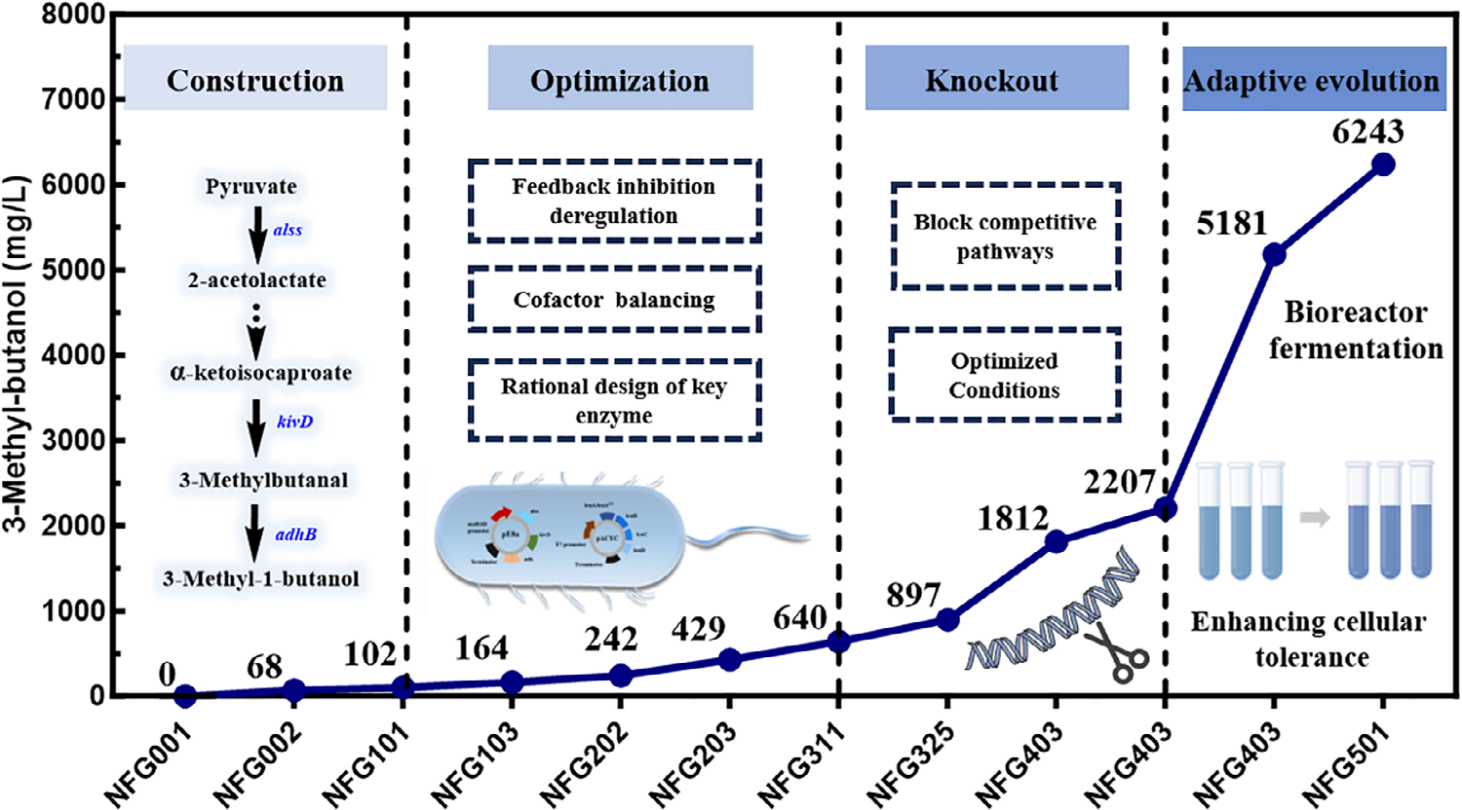
Optimization of the combination strategy for the synthesis of 3‐MB. All cells were cultured according to the experimental procedures.

3‐Methyl‐1‐butanol (3‐MB) is a valuable organic chemical feedstock characterized by its high added value and increasing market demand. However, the non‐fermentative synthesis of 3‐MB not only perturbs cellular metabolism by competing for pyruvate and altering reducing equivalent flux, but is also constrained by product toxicity and the emergence of newly identified rate‐limiting steps, ultimately destabilizing overall metabolic balance. To address these challenges, we undertook a comprehensive optimization and systematic modification of the 3‐MB biosynthetic pathway in the traditional host, *E. coli*. This effort culminated in the development of a fully optimized 3‐MB production pathway that enables cells to synthesize 3‐MB stably and efficiently (Figure 8).

Initially, we engineered strain NFG101 by introducing the heterologous genes *kivD* and *adh2*, achieving a titer of 102.07 mg/L. Subsequent screening of alcohol dehydrogenases (ADHs) led us to *adhB* from *Z. mobilis*, which demonstrated improved activity, increasing the 3‐MB titer to 0.16 g/L (Figure 2b). To further increase carbon flux toward 3‐MB, we overexpressed the *leuABCD* operon, enhancing the conversion of α‐ketoisovalerate (KIV) to α‐ketoisocaproate (KIC). This modification in strain NFG202 led to a 47.3% increase in 3‐MB production. Introducing a feedback‐insensitive *leuA^GT^* (G462D) variant further bypassed metabolic inhibition, resulting in a 67.6% increase, with strain NFG203 reaching 0.43 g/L (Figure 2e).

However, further attempts to enhance flux by overexpressing *ilvC* and *ilvD* led to unexpected drops in titer down to 0.18 g/L in strain NFG301, accompanied by strain instability, likely due to imbalances in redox cofactors. In microaerobic conditions, NADPH synthesis is constrained, while higher alcohol production consumes NADPH, leading to a possible shortage. This, coupled with excess NADH, can disrupt cellular metabolism and growth, highlighting the need for a balanced redox state. To address the redox imbalance, we explored strategies to optimize NADH/NADPH ratios, ultimately replacing NADPH‐dependent *ilvC* with NADH‐dependent *ilvC^BT^* (A71S:R76D:S78D:Q110V). This modification successfully alleviated the growth inhibition and boosted cell biomass by 62%. The corresponding 3‐MB production increased by 117.6% to 0.40 g/L (Figure 3), suggesting that redox management is essential for maintaining cell health and ensuring stable production.

Another major bottleneck was the accumulation of KIC, a key intermediate to 3‐MB (Figure 3d). By fine‐tuning gene expression through dual‐promoter systems, we managed to improve the efficiency of KIC utilization, achieving an 8.4‐fold increase in 3‐MB production compared to strain NFG002, reaching 0.64 g/L in strain NFG311. Notably, *ilvD*, which encodes dihydroxy acid dehydratase, emerged as a key enzyme in this pathway. Rational design and site‐directed mutagenesis of *ilvD* ^ST^ (A268D) improved the catalytic efficiency of *ilvD*, leading to the titer by 40.1%, ultimately reaching 0.90 g/L (Figure 4b).

Molecular dynamics simulations revealed that the A268D mutant DHAD exhibits more restricted structural fluctuations compared to the wild type, resulting in greater conformational stability and tighter receptor–substrate binding. This stability is reflected in lower RMSD values, smaller fluctuations in key atomic distances, a more robust hydrogen‐bonding network, and a global reorganization of the protein's dynamic network. These features collectively contribute to the mutant's enhanced catalytic activity and stability. Additionally, RMSF and DCCM analyses highlight significant local conformational changes and long‐range correlated motions induced by the mutation.

In addition, we targeted competing pathways and knocked out genes including *fnr*, *ldhA*, *mgsA*, *pflB*, and *tdcE*, which are involved in lactate and formate production. These deletions effectively reduced byproduct accumulation and redirected carbon flux toward the desired product. As a result, the engineered strain NFG403 achieved the highest titer of 1.81 g/L, representing a 25.6‐fold increase compared to the parental strain NFG002 (Figure 5b). However, further elimination of byproduct‐associated genes led to a marked inhibition of cell growth, suggesting that excessive disruption of side pathways may impose a significant metabolic burden or disturb cellular regulatory networks, ultimately compromising growth and metabolic capacity. These findings indicate that complete elimination of byproduct formation is not necessarily the optimal strategy in metabolic engineering. Instead, our approach emphasizes the importance of balancing carbon flux redistribution with cell viability to achieve improved overall production performance. Under optimized fermentation conditions, we achieved a peak titer of 2.20 g/L, corresponding to a yield of 0.16 g/g glucose, which represents 48% of the theoretical yield (Figure 5c). However, during microbial fermentation, exposure of cells to higher alcohols results in the generation of reactive oxygen species (ROS), which trigger oxidative stress response cascades and lead to severe growth defects [36].

By employing adaptive laboratory evolution (ALE), we obtained the robust strain NFG501, which sustained stable growth under 5 g/L 3‐MB challenge and demonstrated extended production longevity in bioreactor cultivation. This strain achieved a final titer of 6.24 g/L, representing a 20.5% improvement over the engineered reference strain NFG403 and constituting the highest 3‐MB titer reported to date (Figure 7c and Table 2). Genome analysis indicates that the enhanced tolerance of NFG501 likely arises from system‐level adaptations, including metabolic pathway reprogramming and modulation of post‐transcriptional regulatory networks. By linking specific InDel mutations to functional disruption and system‐level adaptation, this work highlights actionable genetic targets that can be leveraged to rationally enhance microbial tolerance and productivity under industrially relevant solvent stress. These adaptations appear to optimize proteostasis and sustain metabolite flux under solvent stress, providing mechanistic insights and strategic targets for the rational engineering of robust, high‐yielding microbial strains.

In conclusion, we established a globally optimized metabolic engineering strategy for the high‐level production of 3‐methyl‐1‐butanol (3‐MB), carefully balancing cellular growth with product formation. Together, these strategies establish a robust, scalable, and industrially relevant microbial platform for 3‐MB biosynthesis. Further refinement of downstream processing and the exploration of co‐production with branched‐chain derivatives could enhance both the economic viability and industrial applicability of this system, advancing sustainable and efficient microbial production of high‐value alcohols.

## 4 Methods

### 4.1 Strains and Plasmid Construction

Custom DNA oligonucleotide primers were synthesized by GENEWIZ and are listed in Table S1. The plasmids and strains employed in this study are detailed in Tables S2 and S3, respectively. *E. coli* Trans10 (TransGen Biotech Co., Ltd., Beijing, China) was employed for plasmid construction and maintenance, while *E. coli* BL21 (DE3) (TransGen Biotech Co., Ltd., Beijing, China) served as the host strain for metabolic engineering experiments. PCR amplification of target DNA sequences and vector backbone linearization were performed using TransStart FastPFU DNA polymerase (TransGen Biotech). DNA gel extraction, plasmid purification, and fragment assembly were carried out using commercially available kits, including the Gibson Assembly Cloning Kit, all obtained from Vazyme Biotech Co., Ltd. (Nanjing, China). All heterologous genes were codon‐optimized and synthesized by GENEWIZ [37]. The synthesis of key enzyme mutant genes and the construction of expression plasmids are described in the Supporting Information. Unless otherwise specified, all chemical reagents and analytical standards used in this study were of domestic analytical or chemical grade.

### 4.2 Culture Media and Conditions

Lysogeny broth (LB) medium containing 5 g/L yeast extract, 10 g/L tryptone, and 10 g/L NaCl was used for seed propagation, protein expression and feeding experiment. M9‐MOPS medium containing 20 g/L glucose, 5 g/L yeast extract, 11.3 g/L M9 medium, 15.7 g/L MOPS (3‐morpholine propane sulfonic acid), 0.0371 g/L (NH_4_)_6_Mo_7_O_24_·4H_2_O, 0.2431 g/L H_3_BO_3_, 0.0714 g/L CoCl_2_·6H_2_O, 0.0374 g/L CuSO_4_·5H_2_O, 0.1583 g/L MnCl_2_·4H_2_O, and 0.0288 g/L ZnSO_4_·H_2_O was used for de novo synthesis experiments in shake flasks.

For the cell culture experiment, single fresh colonies were inoculated into 5 mL of LB media and grown overnight at 37°C. For the fermentation experiment, the strain BL21(DE3) harboring pE8a‐based and pACYC‐based plasmids was cultivated in 5 mL LB medium overnight at 37°C. Subsequently, overnight cultures (0.5 mL) were transferred to a 250 mL shaking flask containing 50 mL LB media supplemented with 100 µg/mL ampicillin and 25 µg/mL chloramphenicol. The inducer arabinose (L‐ara) and isopropyl‐β‐D‐thiogalactoside (IPTG) were added when the cell OD reached 0.6–0.8. Samples were taken after 48 h and analyzed by GC and HPLC. Cell growth was monitored by measuring the optical density at 600 nm (OD_600_) [38].

### 4.3 Construction of Genetically Defective Strains

A series of gene‐deficient *E. coli* were obtained by knockdown of the *fnr* gene, an important regulator mediating the cellular respiratory system in *E. coli* based on CRISPR/Cas9 gene editing technology, followed by single or a combination of knockdowns of *ldhA*, *mgsA*, *pflB*, *tdcE*, *pta*, *poxB*, *ilvE*, *tyrB*, and *adhE* genes in the competitive pathway. The N20‐sgRNA fragment and the approximately 2000 bp pEcgRNA backbone fragment were amplified separately using the pEcgRNA plasmid as a template with the TransStart Fast PFU DNA polymerase. The targeting plasmid was then constructed by Gibson assembly of the N20‐sgRNA fragment with the backbone fragment. Upstream and downstream homology arms (∼500 bp each) of the target gene were amplified by PCR and joined via overlap extension to generate the complete homology arm fragment. For gene knockout, 400 ng of the homology arm fragment and 100 ng of the targeting plasmid were co‐electroporated into *E. coli* cells carrying the pEcCas plasmid. Cells in which the target gene was successfully disrupted were identified after loss of the plasmid [39]. The required primers are listed in Table S1.

### 4.4 Enzymatic Activity Assay

Dihydroxyacid dehydratase activity: The 200 µL reaction mixture contained 20 mM 2,3‐dihydroxy isovalerate, 10 mM MgCl_2_, 10 mM FeSO_4_, 10 mM L‐cysteine, 0.5 mM 1,4‐dithiothreitol (DTT), 50 mM Tris‐HCl buffer (pH 8.0), and 500 ng of purified enzyme. After incubating at 30°C for 20 min, the consumption of 2,3‐dihydroxyisovalerate was measured by HPLC to determine enzyme activity. One unit of enzyme activity was defined as the amount of enzyme required to consume 1 µM 2,3‐dihydroxy isovalerate per minute.

### 4.5 Laboratory Evolution and Screening of Tolerant Strains

*E. coli* was cultured in M9‐MOPS medium containing 3‐MB, starting at 1  g/L, and subjected to serial passaging with stepwise increases in 3‐MB concentration, ultimately obtaining strains tolerant to 5  g/L. Single colonies were isolated by dilution plating and inoculated into M9‐MOPS medium containing 5  g/L 3‐MB. Cultures were incubated at 37°C and 200 rpm for 24  h, and OD_600_ measurements were used to select strains with enhanced tolerance.

### 4.6 Genome Analysis

Wild‐type and 3‐MB‐tolerant *E. coli* strains were selected and separately cultured to the mid‐logarithmic growth phase. The cultures were harvested by centrifugation at 3000 × g for 10 min at 4°C. The resulting cell pellets were washed twice with pH 7.4 PBS buffer, flash‐frozen in liquid nitrogen, and then submitted to Majorbio for analysis.

### 4.7 Spot Assay

Start by taking 1 mL of bacterial solution. Wash the bacteria twice with sterile PBS (phosphate‐buffered saline), then resuspend the final pellet in 1 mL of sterile PBS. Next, perform serial dilutions to create bacterial solutions with concentrations of 10^−1^, 10^−2^, 10^−3^, 10^−4^, and 10^−5^. Pipette 1.8 µL of each diluted solution onto solid screening media plates containing 3‐MB at different concentrations (1, 2, 3, 4, and 5 g/L). Finally, incubate the plates at 37°C for 12 to 24 h and observe for bacterial growth [40].

### 4.8 Fed‐Batch Fermentation

To expand the production of the strain, fermentation was carried out in a 5 L bioreactor with a 2 L working volume. The culture medium containing 80 g/L glucose, 18 g/L yeast extract, 11.3 g/L M9 medium, 31.4 g/L MOPS (3‐morpholine propane sulfonic acid), 0.01 g/L ampicillin, 0.0025 g/L chloromycetin, 0.0742 g/L (NH4)_6_Mo_7_O_24_·4H_2_O, 0.4862 g/L H_3_BO_3_, 0.1428 g/L CoCl_2_·6H_2_O, 0.0748 g/L CuSO_4_·5H_2_O, 0.3166 g/L MnCl_2_·4H_2_O, and 0.0576 g/L ZnSO_4_·H_2_O was used for fed‐batch fermentation. A single colony was first inoculated into 5 mL of LB medium containing the appropriate antibiotic and cultured at 37°C and 200 rpm for 16 h to generate a preculture. This preculture was then transferred at a 1% inoculum to 100 mL of fresh LB medium with the same antibiotic, and grown at 37°C and 200 rpm until the OD_600_ reached 0.8, serving as the seed culture. The 100 mL seed culture was then inoculated into a fermenter and cultured for 4 h at 37°C with an aeration rate of 1 vvm and a stirring speed of 600 rpm. Following this, induction was initiated by adding arabinose (L‐ara) to a final concentration of 10 mM and isopropyl‐β‐D‐thiogalactoside (IPTG) to a final concentration of 0.5 mM. After 8 h of induction, aeration was stopped and samples were collected at timed intervals [41].

### 4.9 GC and HPLC Analysis

Determination and quantification of higher alcohols. The GC program for higher alcohols quantification was as follows: Detector temperature 210°C, column temperature 60°C for 2 min, 25°C/min to 160°C for 1 min, 10°C/min to 200°C for 1 min, split ratio 30. The by‐products, such as acetic acid, lactic acid, formic acid, etc., were detected by HPLC. For HPLC analysis, High Performance Liquid Chromatography (Agilent Technologies Inc., California, USA) with the Aminex HPX‐87H column (Bio‐Rad Inc., CA, USA), the column temperature was set to 60°C. Phase A was 5 mM dilute sulfuric acid. The following gradient was set as flow rate 0.6 mL/min: 100% phase A for 25 min. All products were quantified based on their specific wavelength and peak area [40].

### 4.10 Molecular Dynamics Simulation

The protonation state of the substrate was examined using Avogadro 1.2.0 software, and a deep computational analysis was performed using ORCA 6.0.1 quantum chemical software. The single‐point energy calculation was performed using the B3LYP functional combined with the D3 dispersion correction and def2‐TZVP basis set to obtain more precise energy information. During the molecular parameterization process, the RESP2 charge of the cyclic dipeptide molecule was calculated using the Multiwfn_3.8_dev software. The ALA268‐ASP mutant protein was constructed with the help of PyMOL, and the FE2‐S2 cluster was constructed using the easyPARM tool (https://github.com/Abdelazim‐Abdelgawwad/easyPAR), where the topological parameters were generated based on the wave function and vibrational data calculated by DFT using the Seminario method and were verified by kinetic tests, and the charge was fitted using the RESP method. Molecular dynamics simulations were performed using the TIP3P water model to simulate the solvent environment and the Na/Cl^−^ ion equilibration. The energy optimization included two consecutive steps: 5000 steps of the steepest descent gradient descent method and 2500 steps of the conjugate gradient method to fully relax the simulation system. During the simulation, the time step was set to 2.0 fs, and the long‐range electrostatic interactions were calculated using the particle mesh Ewald method with 10 Å cutoff. Molecular dynamics simulations of the NPT ensemble were performed at a temperature of 298 K using the Parrinello‐Rahman coupling algorithm, with a simulation step of 2 fs and a total simulation time of 100 ns. Trajectory analysis was performed based on the MDAnal 2.4.0 core package, combined with Python libraries such as NumPy 1.24.3, Pandas 2.0.3, Matplotlib 3.7.2, and SciPy 1.11.1, which systematically analyzed the molecular dynamics simulation data.

The binding free energy between the substrate and protein was evaluated using the gmx_MMPBSA method [42]. Considering the convergence of the trajectory, the last 25 ns of the molecular dynamics simulation was extracted for the binding energy calculations. The MM‐PBSA (Molecular Mechanics Poisson‐Boltzmann Surface Area) approach was employed to decompose the binding free energy into individual energy components, including van der Waals interactions, electrostatic interactions, polar solvation energy, and non‐polar solvation energy. The binding free energy calculations were performed using the gmx_MMPBSA 1.6.2 tool, which provides a comprehensive analysis of protein‐ligand interactions by combining molecular mechanics energies with continuum solvation models. The convergence of the binding energy calculations was monitored throughout the analysis to ensure reliable results.

### 4.11 Statistical Analysis

GraphPad Prism 10.1.2 software was used for data processing. One‐way analysis of variance (ANOVA) with Dunnett's multiple comparison tests was used for the comparison of more than two groups. All values and error bars represent mean and SD (n = 3), respectively, and differences between two groups were compared with a two‐tailed t‐test. Statistical significance was accepted for values of p<0.1.

## Author Contributions

N.G. contributed to experimental design and conduct, data curation, wrote the original draft, wrote, review and edited the final manuscript. H.L. contributed to the simulation calculation and data collection. H.H. and M.M. assisted with the relevant experiments. S.W. contributed to experimental design, writing revision and funding acquisition. T.T. contributed to guidance. H.S. contributed to supervision, resources, funding acquisition, wrote, review and edited the final manuscript.

## Conflicts of Interest

The authors declare no conflicts of interest.

## Supporting information




**Supporting File**: advs74227‐sup‐0001‐SuppMat.docx.

## Data Availability

The data that support the findings of this study are available from the corresponding author upon reasonable request.
