# Supplementary material for: Systematically Engineering for Efficient Production of 3‐Methyl‐1‐Butanol in Escherichia coli
Source: Adv Sci (Weinh). 2026 Feb 12;13(21):e20521. doi: 10.1002/advs.202520521 (PMC13073303; doi:10.1002/advs.202520521)
Supplement: Supplementary file 1 — Supporting File: advs74227‐sup‐0001‐SuppMat.docx. [file ADVS-13-e20521-s001.docx]

**6Supplementary materials**

**Systematically engineering for efficient production of 3-methyl-1-butanol in *Escherichia coli***

Nanfei Geng, Hao Liu, Haolin Han, Meng Meng, Shaojie Wang*, Tianwei Tan, and Haijia Su*

State Key Laboratory of Green Biomanufacturing, National Energy R&D Center for Biorefinery, Beijing Key Laboratory of Green Chemicals Biomanufacturing, Beijing Synthetic Bio-manufacturing Technology Innovation Center, Beijing University of Chemical Technology, Beijing 100029, People’s Republic of China

-

*Corresponding authors:

Haijia Su, E-mail: suhj@mail.buct.edu.cn, Mailing address: State Key Laboratory of Green Biomanufacturing, Beijing University of Chemical Technology, Beijing 100029, People’s Republic of China

Shaojie Wang, E-mail: wangshaojie@buct.edu.cn, Mailing address: State Key Laboratory of Green Biomanufacturing, Beijing University of Chemical Technology, Beijing 100029, People’s Republic of China

**Methods**

**Construction of plasmids for the 3-MB synthesis pathway**

Codon-optimized synthetic genes of *alss*, *kivD*, *adh2*, *adhA* and *adhB* were amplified by primer pairs alss-F/alss-R, kivD-F/kivD-R, adh2-F/adh2-R, adhA-F/adhA-R, and adhB-F/adhB-R, respectively. The plasmid pE8a was used as a template, and the linear plasmid framework was obtained by paB-F/paB-R amplification with primers. Plasmids pE01-pE04 were constructed by ligating the backbone and gene fragments, respectively, according to the Gibson ligation method. pE05 and pA01 according to the same methodology. The required primers are listed in Supplementary Table S2.

**Synthesis of key enzyme mutant genes**

Using *E. coli* endogenous gene *ilvD* as a template, the upstream and downstream fragments of *ilvD* gene were amplified by primer pairs ilvD-F/T1-R and T1-F and ilvD-R, respectively, and overlapped by PCR to obtain the *ilvD^C195K^* gene fragment, and the same method was used to obtain the *ilvD^M85L^*, *ilvD^L164I^*, *ilvD^F199T^*, and *ilvD^A268D^*, and the *ilvC^BT^* and *leuA^GT^* fragment was constructed in the same way, and the expression vector containing the mutant gene was obtained by linking the mutant gene to the gene fragments of the pE8a backbone, *alss*, *kivD*, and *adhB* by Gibson ligation. The pE06-pE14 and pA02 plasmid was obtained as above. The required primers are listed in Supplementary Table S2.


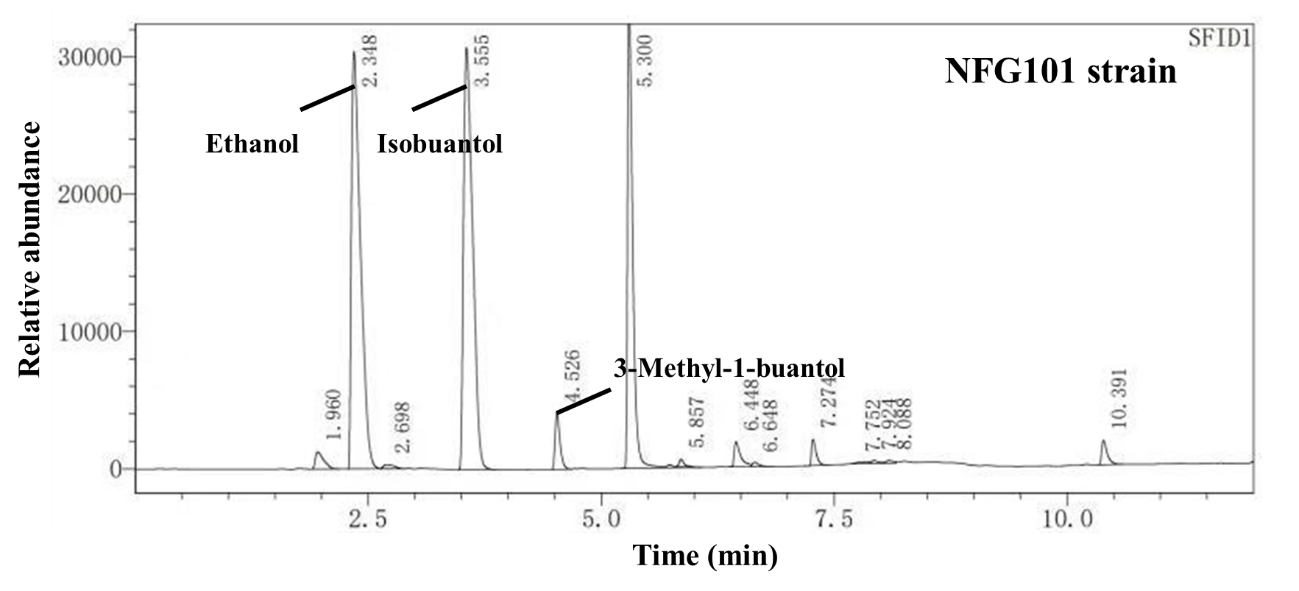


Supplementary Fig. S1 Ethanol, isobutanol and 3-methyl-1-butanol chain-length profiles of strain NFG101. The product was identified by comparing the retention time with the standard sample, and the yield was calculated by the external standard method. All cells were cultured according to the experimental procedures.

Supplementary Fig. S2 Growth of engineered *E. coli* strains NFG001, NFG002, NFG101, NFG102 and NFG103. Each experiment was independently performed at least twice, and representative measurements from three biological replicates are presented as mean ± s.d. All cells were cultured according to the experimental procedures.

Supplementary Fig. S3 Glucose excess of engineered *E. coli* strains NFG001, NFG002, NFG101, NFG102 and NFG103. Each experiment was independently performed at least twice, and representative measurements from three biological replicates are presented as mean ± s.d. All cells were cultured according to the experimental procedures.


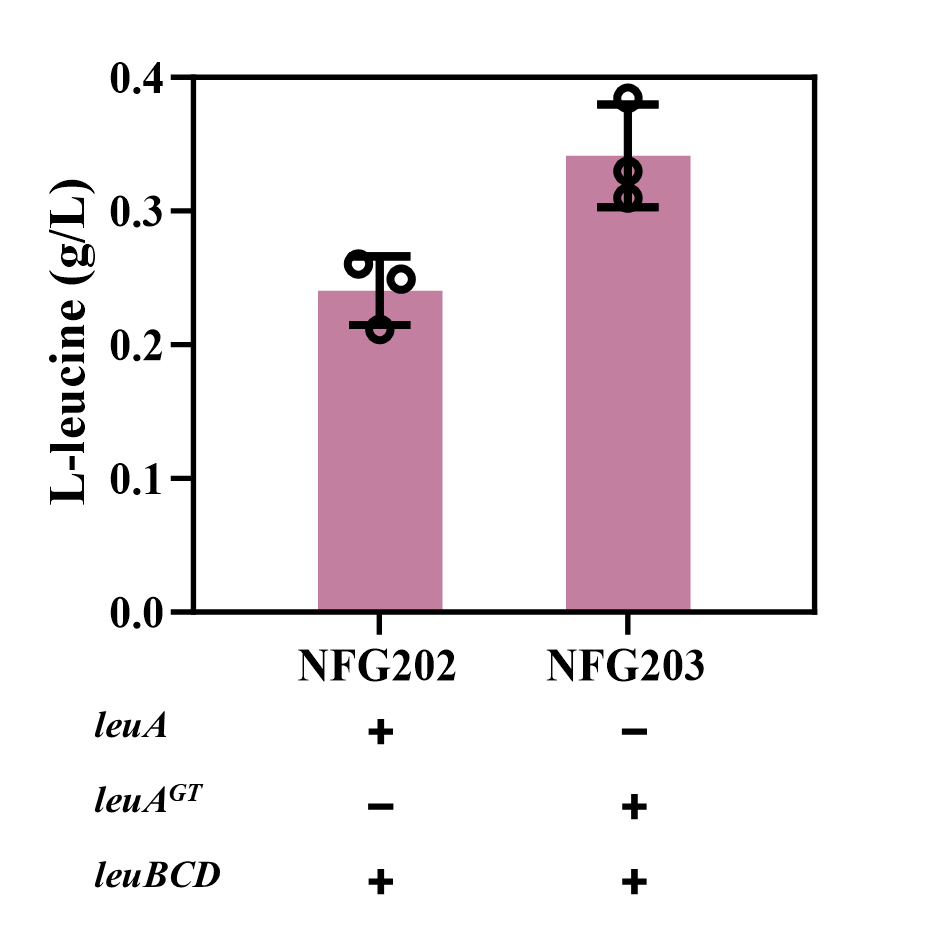


Supplementary Fig. S4 The accumulation of L-leucine in *E. coli* NFG202 and NFG203. Each experiment was independently performed at least twice, and representative measurements from three biological replicates are presented as mean ± s.d. All cells were cultured according to the experimental procedures.

Supplementary Fig. S5 Glucose excess of engineered *E. coli* strains NFG201, NFG202 and NFG203. Each experiment was independently performed at least twice, and representative measurements from three biological replicates are presented as mean ± s.d. All cells were cultured according to the experimental procedures.

Supplementary Fig. S6 Glucose excess of engineered *E. coli* strains NFG301, NFG302, NFG311 and NFG312. Each experiment was independently performed at least twice, and representative measurements from three biological replicates are presented as mean ± s.d. All cells were cultured according to the experimental procedures.

Supplementary Fig. S7 Growth of engineered *E. coli* strains NFG311, NFG321, NFG322, NFG323, NFG324 and NFG325. Each experiment was independently performed at least twice, and representative measurements from three biological replicates are presented as mean ± s.d. All cells were cultured according to the experimental procedures.

Supplementary Fig. S8 Byproduct accumulation of the strain NFG 325. Each experiment was independently performed at least twice, and representative measurements from three biological replicates are presented as mean ± s.d.

Supplementary Fig. S9 Effects of knockout of competing pathways on byproduct accumulation in *E. coli* NFG401 and NFG403. Each experiment was independently performed at least twice, and representative measurements from three biological replicates are presented as mean ± s.d.


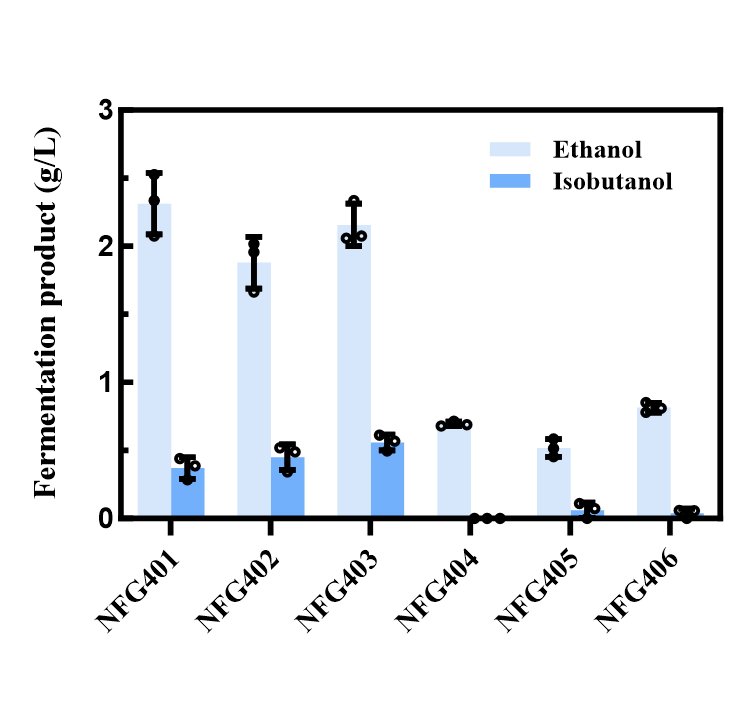


Supplementary Fig. S10 The accumulation of ethanol and isobutanol in *E. coli* NFG401-406. Each experiment was independently performed at least twice, and representative measurements from three biological replicates are presented as mean ± s.d.


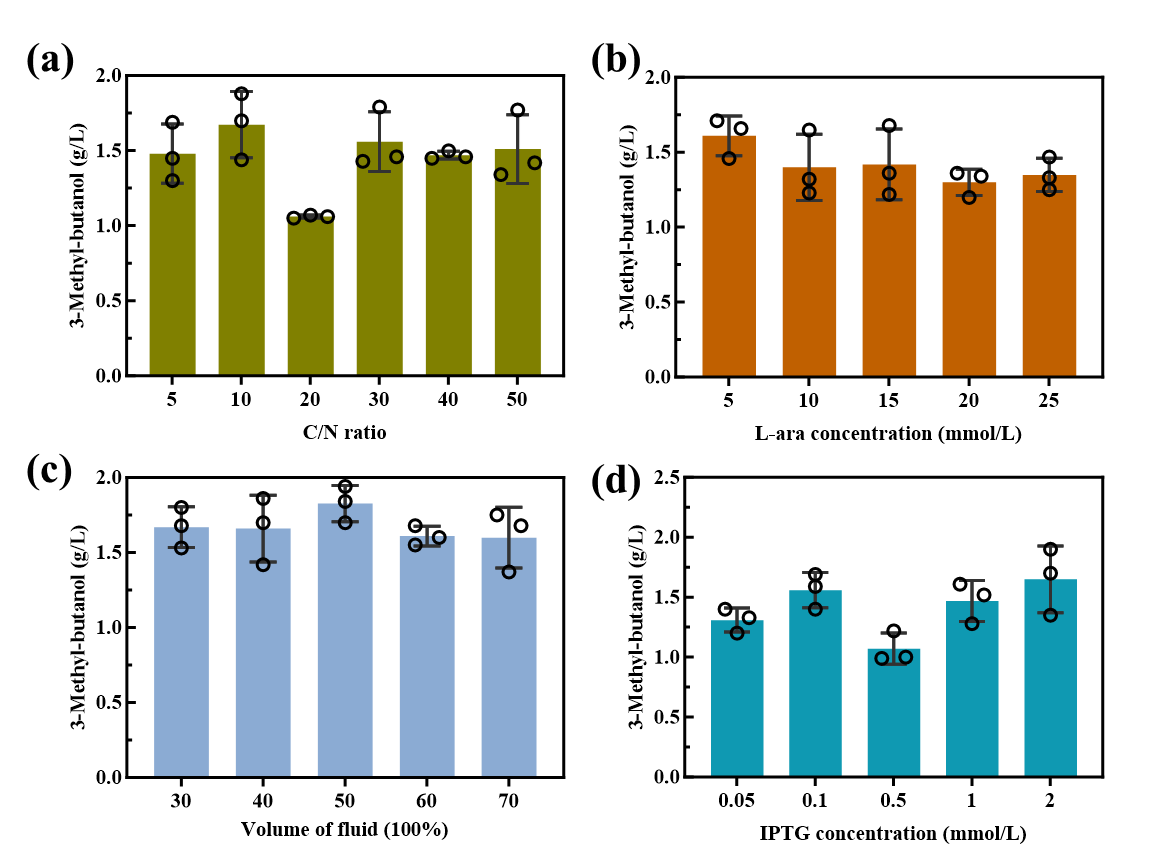


Supplementary Fig. S11 Optimization of fermentation conditions to improve 3-MB production in engineered *E. coli* NFG403. (a) 3-MB production under different C/N conditions. (b) 3-MB production under different L-ara inducer concentrations. (c) 3-MB production under different IPTG inducer concentrations. (d) 3-MB production under different liquid volumes. Each experiment was independently performed at least twice, and representative measurements from three biological replicates are presented as mean ± s.d. All cells were cultured according to the experimental procedures.


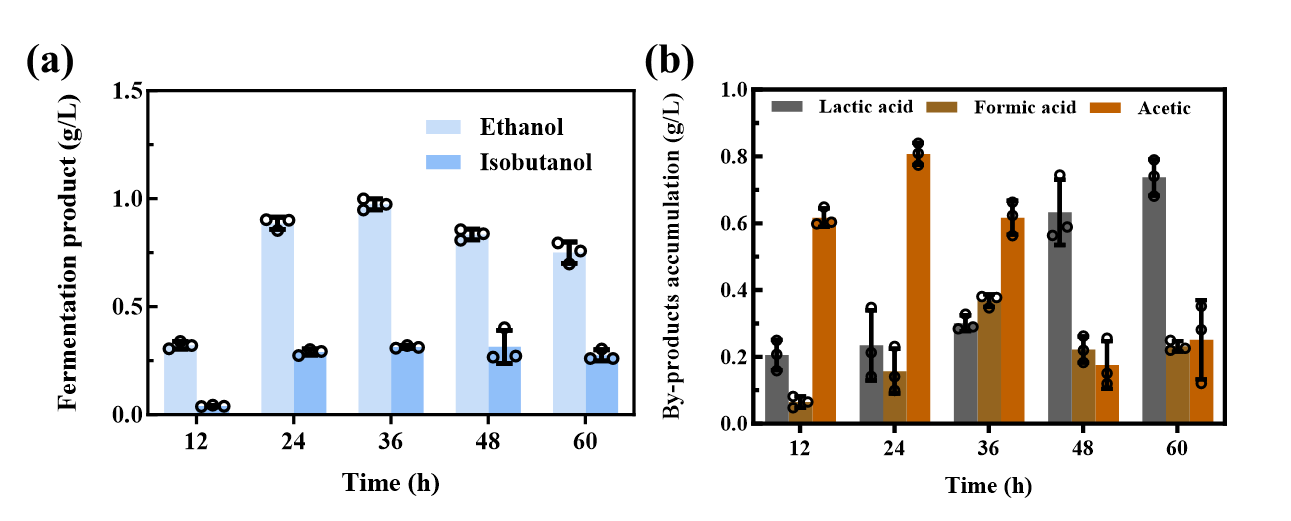


Supplementary Fig. S12 By-product accumulation of engineered *E. coli* NFG403 during 3-MB production under optimized fermentation conditions. (a) The accumulation amount of ethanol and isobutanol. (b) The accumulation amount of lactic acid, formic acid, and acetic. Each experiment was independently performed at least twice, and representative measurements from three biological replicates are presented as mean ± s.d. All cells were cultured according to the experimental procedures.

Supplementary Fig. S13 Cell growth curve of *E.coli* BL21(DE3) under different concentrations of 3-MB stress. We conducted parallel experiments, but in order to better present the comparison effect, only one set of experimental results is shown.


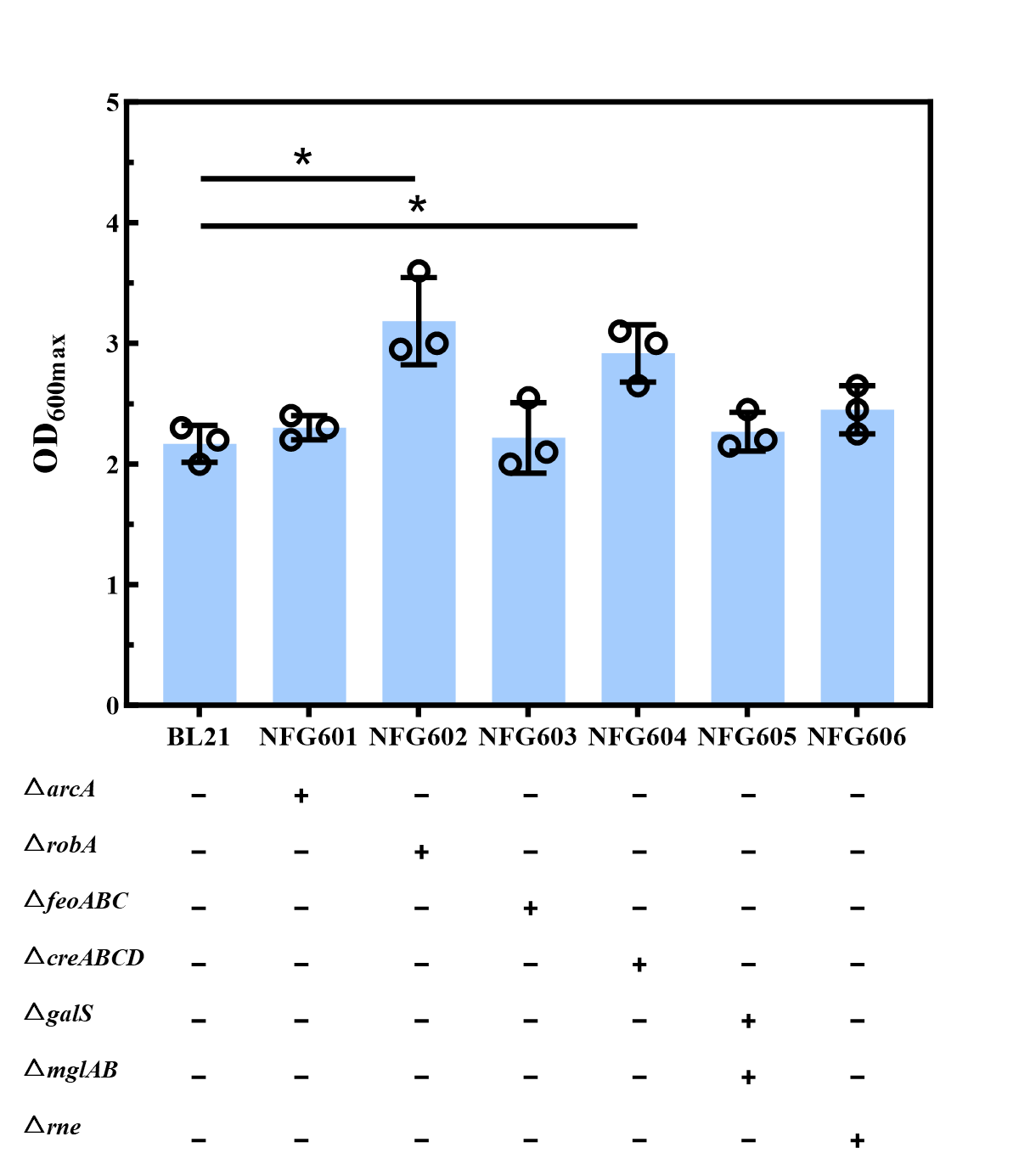


Supplementary Fig. S14 Effect of tolerance-associated gene deletions on the maximum growth of *E. coli* strains NFG601-NFG606. Statistical analysis was performed using t-test (two-tailed, *p＜0.1). Each experiment was independently performed at least twice, and representative measurements from three biological replicates are presented as mean ± s.d.


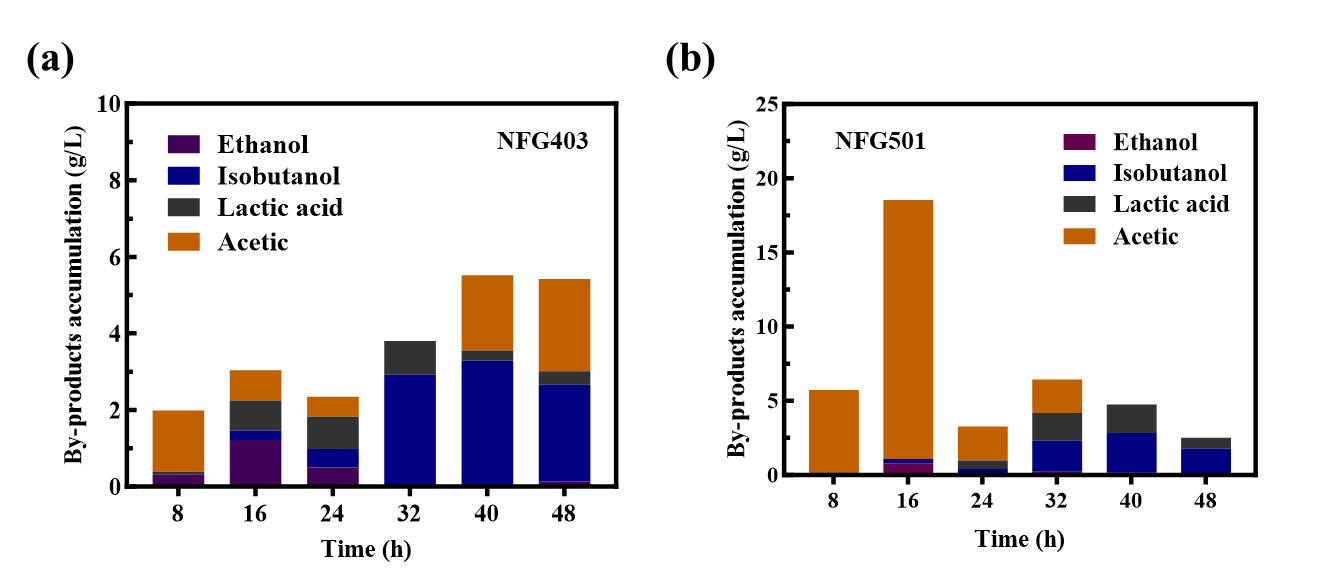


Supplementary Fig. S15 By-product accumulation of engineered *E. coli* strain NFG403(a) and NFG501(b) in a 5L bioreactor.

Supplementary Table S1. Primers used in this study

| Cloning genes | | | | Description | Sequence(5´-3´) |
| --- | --- | --- | --- | --- | --- |
| pE8a-Bone | | | | pB-F | CCAAAGGATCCAAACTCGAGTAAGGATC |
|  |  |  |  | pB-R | TATGTATATCTCCTTCTTAAAAGATC |
| *alss* | | | | alss-F | GATCTTTTAAGAAGGAGATATACATAATGTTGACAAAAGCAACAAAAGAACA |
|  |  |  |  | alss-R | GTATTGAAGTAGTTAGCCATGGTATATCTCCTTCTAGAGAGCTTTCGTTTTCATGAGTTCCCC |
| *kivD* | | | | kivD-F | GGTTAAAAGGAGATATACCATGTATACCGTGGGCGACTATCT |
|  |  |  |  | kivD-R | GGTATATCTCCTTTTAACTTTTGTTCTGCTCGGC |
| *adh2* | | | | adh2-F | CGCCGAGCAGAACAAAAGTTAAAAGGAGATATACCATGAGTATCCCGGAAACCCAG |
|  |  |  |  | adh2-R | TCGAGTTTGGATCCTTTGGTTACTTGCTCGTATCAACCACG |
| *adhA* | | | | adhA-F | ACAAAAGTTAAAAGGAGATATACCATGCGCCTGCAGAACTTTATTTATCGC |
|  |  |  |  | adhA-R | CGAGTTTGGATCCTTTGGTTATTTGGTAAAATCAATCACCATGCGGCCTTCA |
| *adhB* | | | | adhB-F | ACAAAAGTTAAAAGGAGATATACCATGGCGAGCAGCACCTTTT |
|  |  |  |  | adhB-R | CGAGTTTGGATCCTTTGGTTAAAACGCGCTCAGAAACAGTTCTTCCA |
| *ilvC* | | | | ilvC-F | ATGGCTAACTACTTCAATACACTGAATCT |
|  |  |  |  | ilvC-R | TTAACCCGCAACAGCAATACGTT |
| *ilvD* | | | | ilvD-F | AAGGAGATATACCATGCCTAAGTACCGTTCCGCC |
|  |  |  |  | ilvD-R | TGGTATATCTCCTTTTAACCCCCCAGTTTCGATTTATCG |
| *ilvD^C195K^* | | | | T1-F | TGCGGTTCCAAGTCCGGGATGTTTACCGCT |
|  |  |  |  | T1-R | ATCCCGGACTTGGAACCGCAGGTTGGGCA |
| *ilvD^M85L^* | | | | T2-F | TGGGATTGCCCTGGGCCACGGGGGGATG |
|  |  |  |  | T2-R | TGGCCCAGGGCAATCCCATCATCCACCG |
| *ilvD^L164I^* | | | | T3-F | AGCTCGATATCGTTGATGCGATGATCCAGGGC |
|  |  |  |  | T3-R | TCGCATCAACGATATCGAGCTTGATTATCCGATCGG |
| *ilvD^F199T^* | | | | T4-F | GGGATGACCACCGCTAACTCAATGAACTGCC |
|  |  |  |  | T4-R | AGTTAGCGGTGGTCATCCCGGAGCAGGAACC |
| *ilvD^A268D^* | | | | T5-F | CGTTTGAAAACGACATGACGCTGGA |
|  |  |  |  | T5-R | TATCCAGCGTCATGTCGTTTTCAAACG |
| araBAD  promoter | | | | Para-1 | ACGTATTGCTGTTGCGGGTTAACCATTCAGAGAAGAAACCAATTGTCC |
|  |  |  |  | Para-2 | GGTACTTAGGCATGGTATATCTCCTTCCAAAAAAACGGGTATGGAGAAACAG |
|  |  |  |  | Para-3 | ACGTATTGCTGTTGCGGGTTAACCATTCAGAGAAGAAACCAATTGTCC |
|  |  |  |  | Para-4 | GGTACTTAGGCATGGTATATCTCCTTCCAAAAAAACGGGTATGGAGAAACAG |
| pACYC | | | | pACYC-F | AGTCGAACAGAAAGTAATCG |
|  |  |  |  | pACYC-R | ACATGGTATATCTCCTTATTAAAGTTAAAC |
| *leuA/LeuA^GT^* | | | | leuA-F | ATAAGGAGATATACCATGTGGTTTATTCCTCCTATGAGC |
|  |  |  |  | leuA-R | TCACACGGTTTCCTTGTTGTTTTCG |
| *leuB,leuC,leuD（leuBCD）* | | | | leuB-F | AACAAGGAAACCGTGTGATGTCG |
|  |  |  |  | leuD-R | TACTTTCTGTTCGACTTTAATTCATAAACG |
| *△fnr* | | | | fnr-up-F | CAGACGGTCATGTTGGTGC |
|  |  |  |  | fnr-up-R | TGATCCCGGAAGCCTGATTTTTCCGCATAACTCAC |
|  |  |  |  | fnr-down-F | AAAAATCAGGCTTCCGGGATCATAGGTCTGCT |
|  |  |  |  | fnr-down-R | TGCGGGCAGGTAATGCATT |
|  |  |  |  | N20-fnr-1 | CTTTTCTGGAAGCGACCCAG |
|  |  |  |  | N20-fnr-2 | TCAGGCCCAGATAGTTACCG |
|  |  |  |  | N20-fnr-3 | CATGTTCGTTGAGTGTGAAC |
| *△ldhA* | | | | ldhA-up-F | TCCACTTGTGCACCTTCTTTCAG |
|  |  |  |  | ldhA-up-R | GGAGAAAGTCTTAACCCAGGGGAGCTGATTCA |
|  |  |  |  | ldhA-down-F | TCCCCTGGGTTAAGACTTTCTCCAGTGATGTTGAATCAC |
|  |  |  |  | ldhA-down-R | CAGCGGCTGGGATGTGAAA |
|  |  |  |  | N20-ldhA-1 | GCCCGGTAAACAGCACGTTG |
|  |  |  |  | N20-ldhA-2 | GTACCGATAACGCCTGCCGT |
|  |  |  |  | N20-ldhA-3 | GATACGCGCGGTGAATACGG |
| *△mgsA* | | | | mgsA-up-F | ATTGGATGAGAGTCAACTTGACGC |
|  |  |  |  | mgsA-up-R | GACGACTCGCTATTGCACAGGTGGCAAAC |
|  |  |  |  | mgsA-down-F | CTGTGCAATAGCGAGTCGTCAGTTCCATAATG |
|  |  |  |  | mgsA-down-R | GCCTTGAATTGTTGGATGGCG |
|  |  |  |  | N20-mgsA-1 | CACGTCAGGATCGTGCGGCA |
|  |  |  |  | N20-mgsA-2 | CCATTGGGCCACTCAACATC |
|  |  |  |  | N20-mgsA-3 | TAGTGCCTGTTGCATACAG |
| *△pflB* | | | | pflB-up-F | GAGTCTGTTTTGGCAGTCACCT |
|  |  |  |  | pflB-up-R | GGACATCCTGCGTGACCAGAAAGGTGCAGT |
|  |  |  |  | pflB-down-F | TTTCTGGTCACGCAGGATGTCCGGAGTGTAAACG |
|  |  |  |  | pflB-down-R | ATGTCCGAGCTTAATGAAAAGTTAGCC |
|  |  |  |  | N20-pflB-1 | AGTCGGGATAGCGTCACGGT |
|  |  |  |  | N20-pflB-2 | AGACAGGGAGTCAGCAGCAA |
|  |  |  |  | N20-pflB-3 | GATGTACACATCCAAGAAGG |
| *△tdcE* | | | | tdcE-up-F  tdcE-up-R  tdcE-down-F  tdcE-down-R  N20-tdcE-1  N20-tdcE-2  N20-tdcE-3 | TTTTCGAGGCTTAAACGCGCC  CTGCCGTAAATCACCTACGCCAAAGATGG  GCGTAGGTGATTTACGGCAGCGCAGCATA  TTAACGCGCCCGCAGAATT  TAGTCAGAATCGACTGGGTA  GACCTGAATCTTCAGCTTCT  GTGCCGGCCCCATAGTGTGC |
| *△pta* | | | | pta-up-F  pta-up-R  pta-down-F  pta-down-R  N20-pta-1  N20-pta-2  N20-pta-3 | CGGTGCTGTTTTGTAACCCG  CGCTTTGGATTGCGATTTCGTAGTTCAGAGACTG  CGAAATCGCAATCCAAAGCGCCGAACTC  TATCGGGCATTGCCCATCTT  CGTTAACAAACTGAACGCAC  TCGTCTGTCTCCGCCTGCGT  TGTTCTTCATGCTGCTGCCG |
| *△poxB* | | | | poxB-up-F  poxB-up-R  poxB-down-F  poxB-down-R  N20-poxB-1  N20-poxB-2  N20-poxB-3 | TAACGGTAGGGTCGTCTCCGTAAA  AACAAACGGTTGTGGCGATGGAGATGAAAGC  ATCGCCACAACCGTTTGTTTCATGGTTCTC  GTAGAGCAGGAAGTGAAAGCGC  CACCGTTGGCGTACCAACGT  CATCGGTCGGGTAGAAGGCG  CACGATCCGGCACAGACCGC |
| *△adhE* | adhE-up-F  adhE-up-R  adhE-down-F  adhE-down-R  N20-adhE-1  N20-adhE-2  N20-adhE-3 | | | | AGAGGCGTTACAAGTCGGC  AAAAAAGCCCAGCTGGATACCTACTACGGTCGTGA  TAGGTATCCAGCTGGGCTTTTTTTACACGCTCT  AAAGACGCGCTGACAATACG  CCGGTGCGCTCAGACCCAAG  CTGGTCAGTGTACAGGCAAG  CCCATAGCAACCAGTTTCTC |
| *△ilvE* | ilvE-up-F  ilvE-up-R  ilvE-down-F  ilvE-down-R  N20-ilvE-1  N20-ilvE-2  N20-ilvE-3 | | | | GCACTCGACACCATGCTGAA  TGAATACCATCTACGAACCATCTCCCCATTGAACC  GGGAGATGGTTCGTAGATGGTATTCAGGTTGGTGAAGGC  AATAAAGCATCCCCCCGTGG  CTACGACTCGCACAAAGGAC  CCAGCGGGATACTCAACCGA  CCTGGAACCGCGCAGCACCA |
| *△tyrB* | tyrB-up-F  tyrB-up-R  tyrB-down-F  tyrB-down-R  N20-tyrB-1  N20-tyrB-2  N20-tyrB-3 | | | | TTACGCGCCTGACTTCAAGG  TTAACCCGGGCAATGGCATGGCGATAGC  CATGCCATTGCCCGGGTTAAATACGGCAAATGTAC  CTTTGCTGTTTTGCCGAGGAG  CTGGTATGACGAAGCGACTA  CTCTGGTGAGCAATTCGTTC  CTCAGGTTGACCGACTACGT |
| *△**arcA* | arcA-up-F  arcA-up-R  arcA-down-F  arcA-down-R  N20- arcA-1  N20-arcA-2  N20-arcA-3 | | | | CGTGGTATTGGTGTGGTGAAAG  CGTGAACTGCGACACTGGTTCCCAACAA  GAACCAGTGTCGCAGTTCACGCGCTAAC  ATTCACCACGTTTATTAGTTGTATGATGC  CAGAAGCGATAACCTTCACC  CACAGAAGTGAAGCATGGCG  CATCAGGGCCGATCAACGAA |
| *△robA* | robA-up-F  robA-up-R  robA-down-F  robA-down-R  N20-robA-1  N20-robA-2  N20-arcA-3 | | | | GGCTGTGACATCATCTTTGATTCTCG  TCCAAGTGGCATACGGAACGTGCATGCCA  CGTTCCGTATGCCACTTGGAATAACCTGCT  AAATAGCCGCATCGGAGGTATC  TCGTCGTTTGTCGAAATCGG  AATTCACCCAGACGCAGCGG  TTCAGGCCGTAGAGCACTGG |
| *△**feoABC* | | | feoABC-up-F  feoABC-up-R  feoABC-down-F  feoABC -down-R  N20-feoABC-1  N20-feoABC-2  N20-feoABC-3 | | GTGAAGCGCAGCCTGCCG  TGGAGTATGGAGGTGCCTACTTGTTTCTCATTAACTG  AGTAGGCACCTCCATACTCCATCGCCTGTT  TATCCTGGTCCTTGTTCCGTTTT  GTCGTGTGAGCCTGGTATTA  GTTAATACGGTGATAACAAC  ATCCCGCTGGTTTCAACCCG |
| *△**creABCD* | | creABCD-up-F  creABCD-up-R  creABCD-down-F  creABCD-down-R  N20-creABCD-1  N20-creABCD-2  N20-creABCD-3 | | | AGCCACTTGGAATAACCTGCT  CGAATCGCATCAAATAGCCGCATCGGAGG  TGCGGCTATTTGATGCGATTCGCCAAAGTACCA  GGCCAGTTGCTGGTTAAGGTC  TGGCGGCAATAACAGAGGCG  GTTGGTTTGCCTGATATCAG  GTCATCCGTATTGGTCATTT |
| *△galS-mglAB* | | | | SAB-up-F  SAB-up-R  SABdown-F  SAB-down-R  N20- SAB-1  N20-SAB-2  N20-SAB-3 | CGCTGGTTAAGAGAACGCCC  ATTAGCCACCAGGCACTGGAAAATGGTATTTCGAT  TCCAGTGCCTGGTGGCTAATTTCGCCATTGAAG  ATTTATGGATAACATTCCCGGTATGGTGTT  ACGGTGATTGAGAAAGCGCG  TCTTTGTTCAGATCCCAACC  CTTGAGATAAATGCTCGGCG |
| *△rne* | | | | rne-up-F  rne-up-R  rne-down-F  rne-down-R  N20- rne-1  N20-rne-2  N20-rne-3 | TGCCTATTTTGCATTGTTGGTTAGC  TAATGAGCGCCGCAGGTGATTACCGAGTC  ATCACCTGCGGCGCTCATTACGGTCACGG  CTGCGTCTGATCGAAGAAGAAGC  CGCGCAGGTGACGAGGCGAG  GCAAAGCGTAGCCGAAGAAG  GCGGATCCAGACTTTGCCAG |
| pEcgRNA | | | | pEcgRNA-F  pEcgRNA-R | CTGCAGGTCGACTCTAGAGAA  TCTAGAGTCGACCTGCAG |

Supplementary Table S2. Plasmids used in this study

| Plasmids | Description | Source |
| --- | --- | --- |
| pE8a | araBAD promoter, Amp^R^ | Novagen |
| pE01 | pE8a carrying *kivD*, *adh2*, Amp^R^ | This work |
| pE02 | pE8a carrying *alss*, *kivD*, *adh2*, Amp^R^ | This work |
| pE03 | pE8a carrying *alss*, *kivD*, *adhA*, Amp^R^ | This work |
| pE04 | pE8a carrying *alss*, *kivD*, *adhB*, Amp^R^ | This work |
| pE05 | pE8a carrying *alss*, *ilvC*, *ilvD*, *kivD*, *adhB*, Amp^R^ | This work |
| pE06 | pE8a carrying *alss*, *ilvC^BT^*, *ilvD*, *kivD*, *adhB*, Amp^R^ | This work |
| pE07 | pE8a carrying *alss*, *ilvC^BT^*, araBAD promoter, *ilvD*, *kivD*, *adhB*, Amp^R^ | This work |
| pE08 | pE8a carrying *alss*, *ilvC^BT^*, *ilvD*, araBAD promoter, *kivD*, *adhB*, Amp^R^ | This work |
| pE09 | pE8a carrying *alss*, *ilvC^BT^*, araBAD promoter, *ilvD*, *kivD*, *adhA*, Amp^R^ | This work |
| pE10 | pE8a carrying *alss*, *ilvC^BT^*, araBAD promoter, *ilvD^C195K^*, *kivD*, *adhB*, Amp^R^ | This work |
| pE11 | pE8a carrying *alss*, *ilvC^BT^*, araBAD promoter, *ilvD^M85L^*, *kivD*, *adhB*, Amp^R^ | This work |
| pE12 | pE8a carrying *alss*, *ilvC^BT^*, araBAD promoter, *ilvD^L164I^*, *kivD*, *adhB*, Amp^R^ | This work |
| pE13 | pE8a carrying *alss*, *ilvC^BT^*, araBAD promoter, *ilvD^F199T^*, *kivD*, *adhB*, Amp^R^ | This work |
| pE14 | pE8a carrying *alss*, *ilvC^BT^*, araBAD promoter, *ilvD^A268D^*, *kivD*, *adhB*, Amp^R^ | This work |
| pACYCduet-1 | T7 promoters, Cm^R^ | Novagen |
| pA01 | pACYCduet carrying *LeuA*, *LeuB*, *leuC*, *LeuD*, Cm^R^ | This work |
| pA02 | pACYCduet carrying *LeuA^GT^*, *LeuB*, *leuC*, *LeuD*, Cm^R^ | This work |
| pEcgRNA | Derived from pTargetF, ccdB | Laboratory |
| pEcCas | Derived from pCas, sacB, PrhaB-sgRNA-pMB1, pSC101 | Laboratory |
| pEc△fnr | Derived from pEcgRNA, target *fnr* in *E. coli* BL21(DE3) | This work |
| pEc△pflB | Derived from pEcgRNA, target *pflB* in *E. coli* BL21(DE3) | This work |
| pEc△tdcE | Derived from pEcgRNA, target *tdcE* in *E. coli* BL21(DE3) | This work |
| pEc△mgsA | Derived from pEcgRNA, target *mgsA* in *E. coli* BL21(DE3) | This work |
| pEc△ldhA | Derived from pEcgRNA, target *ldhA* in *E. coli* BL21(DE3) | This work |
| pEc△poxB | Derived from pEcgRNA, target *poxB* in *E. coli* BL21(DE3) | This work |
| pEc△pta | Derived from pEcgRNA, target *pta* in *E. coli* BL21(DE3) | This work |
| pEc△adhE | Derived from pEcgRNA, target *adhE* in *E. coli* BL21(DE3) | This work |
| pEc△ilvE | Derived from pEcgRNA, target *ilvE* in *E. coli* BL21(DE3) | This work |
| pEc△tyrB | Derived from pEcgRNA, target *tyrB* in *E. coli* BL21(DE3) | This work |
| pEc△arcA | Derived from pEcgRNA, target *arcA* in *E. coli* BL21(DE3) | This work |
| pEc△robA | Derived from pEcgRNA, target *robA* in *E. coli* BL21(DE3) | This work |
| pEc△feoABC | Derived from pEcgRNA, target *feoABC* in *E. coli* BL21(DE3) | This work |
| pEc△creABCD | Derived from pEcgRNA, target *creABCD* in *E. coli* BL21(DE3) | This work |
| pEc△galS-mglAB | Derived from pEcgRNA, target *galS and mglAB* in *E. coli* BL21(DE3) | This work |
| pEc△rne | Derived from pEcgRNA, target *rne* in *E. coli* BL21(DE3) | This work |

Supplementary Table S3 *E. coli* strains used in this study.

| Strain | Genotype and plasmid | Source |
| --- | --- | --- |
| *E. coli* BL21(DE3) | *E. coli* BL21(DE3) F- ompT hsdSB (rB^–^, mB^–^) gal dcm (DE3) | TransGen Biotech |
| NFG001 | *E. coli* BL21(DE3) carrying pE8a | This work |
| NFG002 | *E. coli* BL21(DE3) carrying pE01 | This work |
| NFG101 | *E. coli* BL21(DE3) carrying pE02 | This work |
| NFG102 | *E. coli* BL21(DE3) carrying pE03 | This work |
| NFG103 | *E. coli* BL21(DE3) carrying pE04 | This work |
| NFG201 | *E. coli* BL21(DE3) carrying pE04 and pACYC-duet | This work |
| NFG202 | *E. coli* BL21(DE3) carrying pE04 and pA01 | This work |
| NFG203 | *E. coli* BL21(DE3) carrying pE04 and pA02 | This work |
| NFG301 | *E. coli* BL21(DE3) carrying pE05 and pA02 | This work |
| NFG302 | *E. coli* BL21(DE3) carrying pE06 and pA02 | This work |
| NFG311 | *E. coli* BL21(DE3) carrying pE07 and pA02 | This work |
| NFG312 | *E. coli* BL21(DE3) carrying pE08 and pA02 | This work |
| NFG313 | *E. coli* BL21(DE3) carrying pE09 and pA02 | This work |
| NFG321 | *E. coli* BL21(DE3) carrying pE10 and pA02 | This work |
| NFG322 | *E. coli* BL21(DE3) carrying pE11 and pA02 | This work |
| NFG323 | *E. coli* BL21(DE3) carrying pE12 and pA02 | This work |
| NFG324 | *E. coli* BL21(DE3) carrying pE13 and pA02 | This work |
| NFG325 | *E. coli* BL21(DE3) carrying pE14 and pA02 | This work |
| NFG401 | *E. coli* BL21(DE3) *△fnr*; carrying pE14 and pA02 | This work |
| NFG402 | *E. coli* BL21(DE3) *△fnr△ldhA△mgsA*; carrying pE14 and pA02 | This work |
| NFG403 | *E. coli* BL21(DE3)*△fnr**△ldhA△mgsA△pflB△tdcE*; carrying pE14 and pA02 | This work |
| NFG404 | *E. coli* BL21(DE3)*△fnr△ldhA△mgsA△pflB△tdcE△pta△poxB*; carrying pE14 and pA02 | This work |
| NFG405 | *E. coli* BL21(DE3)*△fnr△ldhA△mgsA△pflB△tdcE△adhE*; carrying pE14 and pA02 | This work |
| NFG406 | *E. coli* BL21(DE3)*△fnr△ldhA△mgsA△pflB△tdcE△ilvE△tyrB*; carrying pE14 and pA02 | This work |
| NFG501 | *E. coli* BL21(DE3)*△fnr△ldhA△mgsA△pflB△tdcE△ilvE△tyrB*; carrying pE14 and pA02; 180-day adaptive laboratory evolution | This work |
| NFG601 | BL21(DE3)*△arcA* | This work |
| NFG602 | BL21(DE3)*△robA* | This work |
| NFG603 | BL21(DE3)*△feoABC* | This work |
| NFG604 | BL21(DE3)*△creABCD* | This work |
| NFG605 | BL21(DE3)*△galS△mglAB* | This work |
| NFG606 | BL21(DE3)*△rne* | This work |

Supplementary Table S4

| **Production and consumption of cofactors** |
| --- |
| 2-acetolactate + **NADPH** + H^+^→(2R)-2,3-dihydroxy-3-methylbutanoate + NADP^+^  (2R,3S)-3-isopropylmalate + NAD^+^ → α-ketoisocaproate + CO_2_ + **NADH**  3-methylbutanal + **NADPH** + H^+^→3-methyl-butanol + NADP^+^  **acetyl-CoA** + 3-methyl-2-oxobutanoate + H_2_O → (2S)-2-isopropylmalate + coenzyme A + H^+^ |

Supplementary Table S5 Rational design sites and constructed strains of DHAD mutants

|  | | Variants | Mutation Site | Strains |
| --- | --- | --- | --- | --- |
| 1 | *ilvD^C195K^* | | CYS195>LYS | NFG321 |
| 2 | *ilvD^M85L^* | | MET85>LEU | NFG322 |
| 3 | *ilvD^L164I^* | | LEU164>ILE | NFG323 |
| 4 | *ilvD^F199T^* | | PHE199>THR | NFG324 |
| 5 | *ilvD^A268D^* | | ALA268>ASP | NFG325 |

Supplementary Table S6 Binding free energy decomposition analysis using gmx_MMPBSA

| Energy Component | Wild-type DHAD | A268D Mutant | Difference (Δ) |
| --- | --- | --- | --- |
| ΔVDWAALS | -13.49 ± 1.85 | -13.23 ± 3.69 | +0.26 |
| ΔEEL | +25.76 ± 4.78 | -10.86 ± 7.27 | -36.62 |
| ΔGGAS | +12.26 ± 4.56 | -24.09 ± 9.08 | -36.35 |
| ΔEPB | -23.75 ± 3.66 | +12.75 ± 7.07 | +36.50 |
| ΔENPOLAR | -1.84 ± 0.10 | -1.70 ± 0.12 | +0.14 |
| ΔGSOLV | -25.58 ± 3.65 | +11.05 ± 7.01 | +36.63 |
| ΔTOTAL | -13.32 ± 1.84 | -13.04 ± 3.16 | +0.28 |
| ΔG binding | -8.01 ± 2.72 | -14.30 ± 3.16 | -6.29 |

Supplementary Table S7 Mutated gene loci identified by whole-genome resequencing of tolerant bacterial strains

| **Gene** | **Mutation type** | **Gene function** |
| --- | --- | --- |
| *arcA* | insertion mutation | AcrA is the periplasmic lipoprotein component of the AcrAB-TolC and AcrAD-TolC multidrug efflux pumps. |
| *bioH* | deletion mutation | BioH, an esterase, hydrolyzes the methyl ester of pimeloyl-[acp] and terminates the fatty acid synthesis enzyme-catalyzed part of the biotin biosynthesis pathway. |
| *cirA* | insertion mutation | Cir is a member of the Outer Membrane Receptor (OMR) family of porins. Cir is a TonB-dependent iron-siderophore complex uptake receptor. |
| *creA* | insertion mutation | *creA* regulates the expression of the creD-lacZ transcriptional fusion. |
| *creB* | insertion mutation | DNA-binding transcriptional regulator CreB. |
| *creC* | insertion mutation | CreC is the sensor histidine kinase of the CreCB two-component signal transduction system. |
| *creD* | insertion mutation | CreD is a predicted inner membrane protein with five transmembrane domains, and its terminus is cytoplasm-located. |
| *feoA* | deletion mutation | FeoA is part of a conserved ferrous iron transport system. |
| *feoB* | frameshift mutation | FeoB is the inner membrane component of a ferrous iron uptake system which is active under anaerobic growth conditions. |
| *feoC* | deletion mutation | FeoC is part of a conserved ferrous iron transport system. |
| *fhuE* | frameshift mutation | FhuE is a protein which serves as a receptor for ferric-coprogen (a hydroxamate-type iron chelator) and ferric-rhodotorulic acid. |
| *folE* | insertion mutation | *folE* is required for preQ₀ biosynthesis from GTP (deletion eliminates tRNA queuosine ribonucleoside). |
| *galS* | insertion mutation | GalS, is a DNA-binding transcription factor that represses transcription of the operons involved in transport and catabolism of D-galactose. |
| *gntX* | deletion mutation | GntX is thought to be involved in high-affinity gluconate transport. |
| *htrE* | base substitution | An *htrE* insertion mutant exhibits heat sensitivity, HtrE is subject to posttranslational |
| *lysP* | insertion mutation | LysP is a lysine-specific transporter that mediates lysine uptake for biosynthesis and acts as a co-sensor for lysine in the lysine-dependent acid-resistance (LDAR) system. |
| *mglA* | insertion mutation | MglA is the predicted ATP-binding component of a D-galactose / D-galactoside ABC transporter. |
| *mglB* | insertion mutation | MglB is the periplasmic binding protein of the D-galactose/methyl-D-galactoside ABC transport system, and ligand-bound MglB interacts with Trg to mediate taxis to galactose and glucose. |
| *mrcB* | deletion mutation | Penicillin binding protein 1B (PBP1B), the product of the *mrcB* gene, is a bifunctional peptidoglycan synthase with transglycosylase and transpeptidase activity. |
| *nfuA* | deletion mutation | NfuA is a 'non-ISC, non-SUF' Fe-S cluster carrier protein that receives Fe-S clusters from SufBC2D and IscU/HscBA complexes and transfers them to SufA and IscA. |
| *panB* | base substitution | PanB (KPHMT) catalyzes the first committed step in pantothenate biosynthesis, transferring C11 of 5,10-methylene-tetrahydrofolate to 2-keto-isovalerate to form 2-dehydropantoate. |
| *panC* | base substitution | Pantothenate synthetase (PanC) catalyzes the ATP hydrolysis-dependent synthesis of pantothenate from β-alanine and pantoate. |
| *rluC* | deletion mutation | RluC catalyzes pseudouridylation of U955, U2504, U2580 in 23S rRNA (near ribosomal peptidyl transferase center) and has in vitro activity toward 16S rRNA. |
| *rne* | frameshift mutation | RNase E is an essential single-strand-specific endonuclease that processes rRNA, tRNA and other RNAs, participates in plasmid/phage stability, and is part of the degradosome (for mRNA degradation). |
| *robA* | frameshift mutation | DNA-binding transcriptional dual regulator Rob. |
| *rpmF* | deletion mutation | The L32 protein is a non-essential component of the 50S subunit of the ribosome. |
| *rpnA* | deletion mutation | rpnA interacts with Fe-S cluster biosynthesis genes; its mutant has slightly higher stationary-phase cell density than wild type in sublethal streptomycin. |
| *rpnC* | base substitution | RpnC is one of five proteins in *E. coli* that belong to the "transposase_31" family, which is distantly related to the PD-(D/E)XK nuclease superfamily. |
| *rseB* | insertion mutation | RseB, a negative regulator of sigma E activity, interacts with RseA (anti-sigma E factor)–it binds RseA's periplasmic region, stimulates RseA-sigma E binding, and prevents DegS from cleaving RseA. |
| *yceD* | deletion mutation | YceD is a protein of unknown function. |
| *yceF* | deletion mutation | YceF shows triphosphatase activity with the modified nucleotide 7-methyl-GTP (m7GTP) as a substrate. |
| *yeiB* | insertion mutation | No information about this protein. |
| *yeiG* | frameshift mutation | YeiG has significant activity with lactoylglutathione (a methylglyoxal detoxification intermediate); it may be a cytoplasmic equivalent of glyoxalase II, potentially involved in endogenous methylglyoxal detoxification. |
| *yhgF* | deletion mutation | YhgF consists of multiple predicted domains, among them an S1 domain, commonly found in extensively researched RNA-binding proteins (RBPs). |
| *yieE* | insertion mutation | No information about this protein was found. |
